# Trends and spatial distribution of precarious work conditions for nurses in Brazil based on the type of employment bond*

**DOI:** 10.1590/1518-8345.7680.4644

**Published:** 2025-11-17

**Authors:** Érika Carvalho de Aquino, Rafael Alves Guimarães, Daniel do Prado Pagotto, Joseane Aparecida Duarte, Antônio Isidro da Silva, Cândido Vieira Borges

**Affiliations:** 1 Universidade Federal de Goiás, Instituto de Patologia Tropical e Saúde Pública, Goiânia, GO, Brazil.; 2 Universidade Federal de Goiás, Faculdade de Enfermagem, Goiânia, GO, Brazil.; 3 Universidade de Brasília, Brasília, DF, Brazil.; 4 Universidade Federal da Bahia, Salvador, BA, Brazil.; 5 Universidade Federal de Goiás, Faculdade de Administração, Ciências Contábeis e Ciências Econômicas, Goiânia, GO, Brazil.

**Keywords:** Workforce, Work Conditions, Precarious Work, Job Security, Nurses, Health Care Levels

## Abstract

to analyze the tendencies and spatial distribution of precarious work for nurses in Brazil, based on the type of employment relationships, between 2010 and 2023, according to the level of health care.

ecological study with data extracted from the National Registry of Health Establishments. The indicator analyzed was the percentage of precarious work relationships. The temporal tendency was assessed by the Prais-Winsten regression model and the spatial distribution was assessed by means of choropleth maps.

there was an increasing tendency in the precarious work of nurses in Brazil based on the type of employment relationships, regardless of the level of health care. The North region showed the highest percentages of increase in the indicator in Primary Health Care, Secondary Health Care, and Tertiary Health Care. The North and Northeast regions had the highest numbers of municipalities with a high percentage of precarious work conditions.

the precariousness of nursing employment relationships showed an increasing tendency at all levels of health care, being higher in Primary Health Care and increasing more in Tertiary Health Care within the time horizon analyzed.

## Highlights

**(1)** The precariousness of nurses’ employment relationships has increased substantially. **(2)** The precariousness of employment relationships was high in Primary Health Care. **(3)** The North and Northeast regions showed the most precarious employment relationships. **(4)** Tertiary Health Care has seen the greatest increase in the precariousness of employment bonds.

## Introduction

The Precariousness of Employment Relationships (PER) represents a serious global problem and has adverse consequences on the health of workers, management and quality of health care^(1-2)^, reducing the capacity of health systems to achieve their objectives, especially Universal Health Coverage^(3)^. Precarious Work (PW) is a multidimensional construct characterized by low-quality employment conditions, including short fixed-term contracts, temporary contracts, low pay, reduced access to unionization rights and job insecurity^(4-5)^.

In Brazil, PER represents a growing challenge for the Unified Health System (SUS)^1(6)^, which is being exacerbated by neoliberal policies, increased outsourcing, and flexible contracts^(7)^ due to legislative changes and budgetary restrictions in the health system, such as Constitutional Amendments No. 19 of 1998^(8)^ and No. 95 of 2016^(9)^, and the establishment of public and private partnerships to establish Health Care Networks^(7)^. These measures have exacerbated regional inequalities and hindered the allocation of qualified professionals in the territories^(10)^. Studies have shown that flexible contracts, especially outsourcing and freelancing, have resulted in more precarious employment relationships, compromising the quality of services provided by the SUS^(11-12)^.

One of the professional categories working in SUS most impacted by PER is nursing, made up of nurses, nursing technicians and nursing assistants^(2-13)^. Nursing is the largest Healthcare Workforce (HWF) in the country^(14)^, playing a fundamental role in the performance of SUS at all levels of care. Despite this, many nursing professionals face precarious working conditions, such as temporary contracts with no guarantees of social rights, low pay and overload^(15-16)^. This reality compromises the system’s ability to offer quality healthcare services and retain professionals in low-population regions^(16)^.

Despite this, no broad national study with population data has been conducted to assess trends in PER among nurses, especially those disaggregated by regions and states. Furthermore, this phenomenon may behave differently across levels of health care, with Primary Health Care (PHC) encompassing the majority of precarious contracts^(6)^. Therefore, there is also a gap in the literature on trends in PER among nurses according to the level of health care. In view of this scenario, the study of trends and spatial distribution of PER in nurses is essential and emerging in the country, since it is aligned with international agendas, such as the eighth Sustainable Development Goal of the United Nations^(17)^ and the Global Strategy for Human Resources in Health of the World Health Organization^(18)^, in addition to contributing to subsidizing strategies for the reduction of precarious employment relationships in health, such as the National Program for the Reduction of Precarious Employment in SUS^(19)^.

Therefore, the objective of this study was to analyze the trends and spatial distribution of precarious work for nurses in Brazil, based on the type of employment relationship, between 2010 and 2023, according to the level of health care.

## Method

### Study design

Ecological study of time series and population-based type. The reporting of this manuscript was carried out using adaptations of the Revised Standards for Quality Improvement Reporting Excellence (SQUIRE 2.0)^(20)^.

### Context

The study was conducted in Brazil, based on data from all Brazilian municipalities from 2010 to 2023. This period was chosen because it allows for the analysis of a broader historical line, providing support for the discussion of various laws, programs and public policies that contributed to PER in this period. In addition, this time frame includes both the period before and after the COVID-19 pandemic, enabling the assessment of changes in the context of professional relationships during and after the health crisis, in which many nurses were subjected to precarious employment relationships^(12)^.

The country had an estimated population of 203,080,756 million inhabitants in 2022, according to the latest demographic census by the Brazilian Institute of Geography and Statistics, distributed across 5,568 municipalities^(21)^, grouped into 26 federative units and the Federal District. The regions have different demographic, social and economic characteristics, as well as differences in the number of nurses and the number of devices and investments in Health Care Network^(22-23)^.

### Participants

The study included all employment relationships of nurses working in health services in Brazil registered in the National Registry of Health Establishments (NRHE) between 2010 and 2023, regardless of the level of activity (primary, secondary and tertiary).

### Data source and variables

Data on the employment relationships of nurses working in health services in Brazil were extracted from NRHE microdata, in the layout of professional files, which is publicly available. The extraction was performed using the file transfer protocol from the SUS Information Technology Department, on January 10th, 2024.

NRHE is a Health Information System that contains data on the physical structure of health units, available health services and professionals linked to health establishments in the national territory, at all levels of management (national, state and municipal), whether or not linked to SUS. Data are entered by filling out forms with variables on the physical structure, equipment, HWF, among other elements^(24)^.

The following data were extracted from NRHE: the code of the municipality of the health establishment, NRHE code of the unit, type of unit, Brazilian Code of Occupations (BCO), type of professional relationship, and subtype of professional relationship. BCOs whose BCO family started with code 2235, corresponding to nurses and similar, were filtered.

Microdata is the smallest disaggregation of the NRHE, meaning that each observation represents an employment relationship. These were organized according to the type and subtype of relationship, extracted from the field “employment relationship with the establishment”. According to a previous study^(6)^, the relationships were classified as: (i) protected: government employee, government employee assigned to the private sector, *celetista* (employee governed by Brazilian labor law, CLT - Consolidated Labor Laws), public employee and statutory; (ii) precarious: self-employed (including those with a direct relationship, without intermediation or those with an indirect relationship, when intermediated by institutions or entities such as cooperatives, Civil Society Organizations of Public Interest, philanthropic and/or non-profit entities, private companies, Non-Governmental Organizations and Social Organizations), scholarship holders, cooperative members, verbal or informal contracts, volunteering, commissioned positions, temporary or fixed-term contracts; (iii) others: owners, interns and residents and (iv) no information: type of employment relationship absent in the NRHE.

Employment relationships were classified according to the level of care as: (i) PHC (6,25), which included the following NRHE codes: 01 – health post; 02 – health center/basic unit; 32 – river mobile unit; 40 – land mobile unit; 71 – family health support center; 72 – indigenous health care unit and 74 – health academy hub; (ii) Secondary Health Care (SHC), which included the following codes: 04 – polyclinic; 15 – mixed unit; 20 – general emergency room; 21 – specialized emergency room; 22 – isolated office; 36 – specialized clinic/center; 39 – diagnostic and therapeutic support unit; 42 – pre-hospital mobile unit in the emergency area; 61 – isolated birth center; 62 – isolated day hospital; 69 – hemotherapy and/or hematologic care center; 70 – CAPS; 73 – emergency care; 83 – disease and injury prevention and health promotion centers and (iii) Tertiary Health Care (THC), which included codes 05 – general hospital and 07 – specialized hospital. Thus, relationships associated with other health establishments were excluded.

Previous studies have also used analyzes on the types of employment relationships to investigate precariousness^(6,11,25)^. However, the classification adopted in this study was chosen because it is categorical in relation to the types of relationships and their respective degrees of precariousness^(6)^, in addition to being compatible with the data structure used, the NRHE.

The indicator analyzed was the percentage (%) of precarious employment relationships of nurses. This indicator was calculated, for each year, using the following formula:

$$\frac{Númerodevínculoslaboralesprecariosendeterminadalocalidadyperíodo}{Númerototaldevínculosenlamismalocalidadyperíodo}x100$$

The time horizon was from 2010 to 2023. The numbers of links referring to the month of June of each year were analyzed, as it is the middle of the year, in addition to there being less influence of management changes in this period and being congruent with annual population indicators that also refer to this month.

### Statistical analysis

Initially, descriptive analyses were performed using the absolute number of PER and the percentage (%) of precarious employment relationships.

Then, temporal trends were analyzed using the Prais-Winsten linear regression model. Before inclusion in the regression models, the base 10 logarithmic transformation of the rates was performed to reduce the heterogeneity of the variance of the residuals and contribute to the calculation of the temporal trend^(26)^. The dependent variable (Y) used was the percentage (%) of precarious employment relationships, while the independent variable (X) was the year of the time series. The regression equation was defined by t ^(26)^, where is the percentage (%) of precarious employment relationships of nurses after the logarithmic transformation, β_0_ is the intercept or regression constant, β_1_ is the slope coefficient of the line and *e_t_* is the random error. The “t” estimates the times of the data set {*t*_1_, ..., *t*_14_}, in the case *t*_1_=2010 and *t*_14_=2023.

Through the regression models, it was possible to obtain the value of the slope coefficient of the line (β_1_)and the standard errors (SE). With these parameters, the Annual Percentage Variation (APV) was calculated, according to the following formula^(26)^:

$$VPA=\left(1+10^{\beta_{1}}\right)*100$$

where β_1_ is the slope coefficient of the line.

The lower and upper limits of the 95% Confidence Interval (95%CI) of the APV were calculated using the formula^(26)^:

$$IC95\%=\left(1+10^{\beta_{1}\left(t*EP\right)}\right)*100$$

where β_1_ is the slope coefficient of the line obtained in the regression model, t is the value that the Student’s t distribution presents with 13 degrees of freedom (n-1) at a two-tailed 95% CI and EP is the standard error of the estimate of β_1_ obtained in the regression.

The analyses were performed in a disaggregated manner according to the level of care: (i) PHC, (ii) SHC and (iii) THC for Brazil, the five major regions and the 27 federative units, constituting 99 time series analyzed. In addition, analyses of the spatial distribution of the indicator were performed according to the level of care for the years 2010, 2016 and 2023. Choropleth maps were constructed, using the number of Brazilian municipalities as the unit of analysis (n=5,568).

Data extraction and processing were performed using Structured Query Language (SQL). Descriptive analyses and time series models were performed using R, with the RStudio interface^(27)^. Spatial distribution analyses were conducted using QGIS software (QGIS Development Team, 2024)^(28)^.

### Ethical aspects

There was no need for submission to the Research Ethics Committee, since exclusively secondary and publicly accessible microdata were used, through the DATASUS file transfer tool, as waived in Resolution No. 510/2016 of the National Health Council^(29)^.

## Results

Between 2010 and 2023, 47,411,575 nursing employment contracts were registered in the NRHE. Of these, 2,629,306 (5.54%) records related to health units whose types were not part of the analysis and therefore, were excluded. Thus, 44,782,269 employment contracts were analyzed, representing 94.46% of the total contracts in the period. Of these, 12,186,668 (27.21%) were contracts in PHC units, 8,836,941 (19.73%) in SHC and 23,758,660 (53.05%) in THC.

**Figure 1-:**
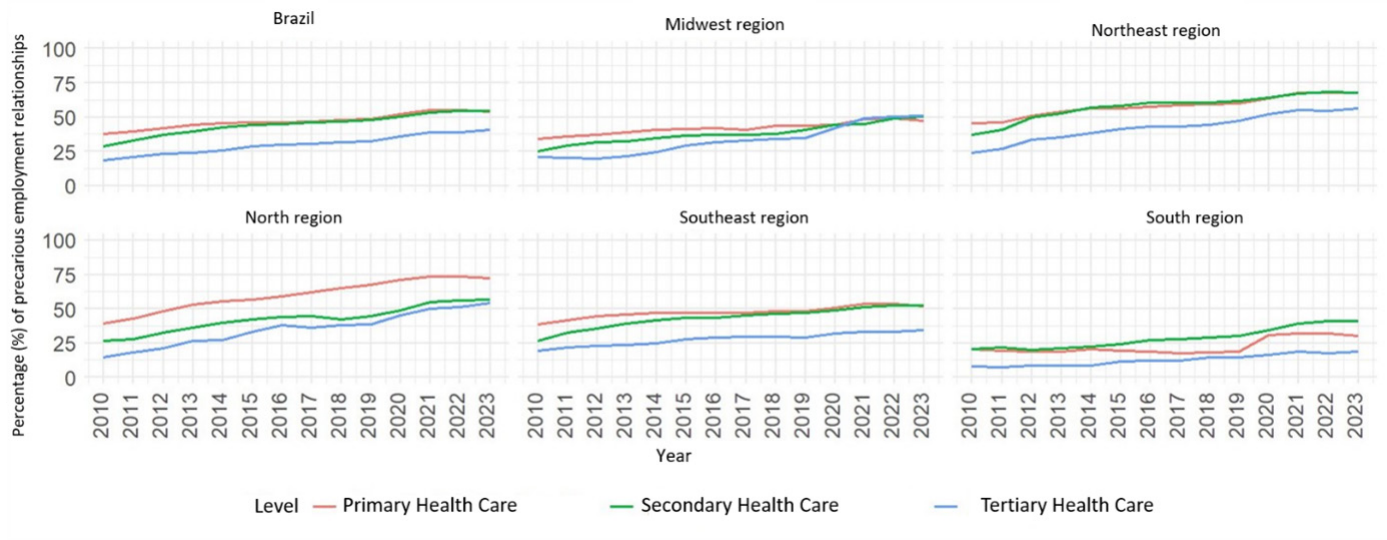
Percentage (%) of precarious employment relationships among nurses, according to level of health care and regions of Brazil, 2010-2023 (n = 44,782,269)

Note: Number of observations (n) included in the analysis of the historical series: (ii.1): Primary Health Care: 12,186,668; (ii.2): Secondary Health Care: 8,836,941 and (ii.3): Tertiary Health Care: 23,758,660

Figure 1 shows the evolution of the percentage (%) of precarious employment contracts for nurses, according to level of care and regions. In Brazil, the percentage of precarious contracts was similar in PHC and SHC and lower in THC. This pattern is similar in the Southeast and Northeast regions. The South region has the highest percentage of precarious employment relationships in SHC when compared to other levels of care, while the North region has the highest percentage in PHC. In the Midwest region, the percentages of precarious employment relationships between levels of care show similarity over time, with similar percentages starting in 2021.

Brazil showed an increasing tendency in the percentage of PER in PHC (APV = 2.8%; 95%CI = 2.1-3.4%). All regions showed an increasing trend in the indicator in PHC, except the South region. The North region showed the highest percentage increase (APV = 4.8%; 95%CI = 3.1-6.6%). Among the federation units, 21 (77.8%) showed an increasing trend, while six (22.2%) showed a stationary trend in the percentage of PER of nurses in PHC (Table 1).

**Table 1-:** Tendency in the percentage (%) of precarious employment relationships for nurses in Primary Health Care, based on the type of relationship, according to Brazilian states and regions, 2010-2023 (n = 12,186,668)

**Regions and Federation Units**	**Number and percentage of precarious employment relationships**							**Prais-Winsten regression**			
	**2010**		**2016**		**2023**		**APC** ^‡^	**CI95%** ^§^		*p* -value**	**Tendency**
	n*	**%** ^†^	n*	**%** ^†^	n*	**%** ^†^		**LL** ^||^	**SL** ^¶^		
**Region North**	1,758	39.1	3,717	59.3	6,468	72.4	4.8	3.1	6.6	<0.001	Increasing
Rondônia	65	14.2	108	17.9	264	33.5	7.6	4.8	10.5	<0.001	Increasing
Acre	58	20.8	124	32.7	211	43.7	6.1	3.6	8.7	<0.001	Increasing
Amazonas	506	53.4	976	72.1	1,730	85.9	3.7	2.8	4.6	<0.001	Increasing
Roraima	96	61.1	152	56.3	267	60.7	0.1	-3.1	3.4	0.947	Stationary
Pará	733	39.6	1,746	67.8	2,744	78.2	5.3	2.7	8.0	0.001	Increasing
Amapá	9	3.6	182	56.0	419	74.3	25.5	7.0	47.1	0.010	Increasing
Tocantins	291	52.7	429	56.0	833	73.9	3.6	2.7	4.5	<0.001	Increasing
**Region Northeast**	7,865	45.4	11,689	57.5	18,128	67.6	3.1	2.4	3.8	<0.001	Increasing
Maranhão	1,376	60.9	1,991	73.4	2,850	82.9	2.3	1.6	3.0	<0.001	Increasing
Piauí	258	21.4	555	36.2	983	49.1	7.0	5.3	8.7	<0.001	Increasing
Ceará	739	27.3	1,684	51.5	2.793	62.4	6.5	4.1	8.9	<0.001	Increasing
Rio Grande do Norte	685	62.4	754	57.9	1,033	63.1	0.5	-0.1	1.0	0.101	Stationary
Paraíba	739	46.6	883	49.9	1,347	59.3	2.3	1.5	3.1	<0.001	Increasing
Pernambuco	1,416	60.0	1,997	69.5	2,560	70.7	1.2	0.3	2.0	0.013	Increasing
Alagoas	240	24.5	391	34.5	997	60.7	7.1	5.4	8.7	<0.001	Increasing
Sergipe	130	18.4	247	31.9	775	63.3	11.1	10.2	12.1	<0.001	Increasing
Bahia	2,282	51.8	3,187	64.1	4,790	73.6	2.6	1.6	3.5	<0.001	Increasing
**Region Southeast**	7,675	38.7	12,451	47.1	19,350	52.1	2.1	1.4	2.8	<0.001	Increasing
Minas Gerais	2,857	44.9	4,408	53.2	6,383	55.0	1.5	0.6	2.3	0.004	Increasing
Espírito Santo	496	41.8	565	47.7	1,207	65.5	3.3	1.6	5.0	0.001	Increasing
Rio de Janeiro	1,540	50.3	2,870	62.6	3,880	67.2	1.9	0.8	3.0	0.003	Increasing
São Paulo	2,782	30.2	4,608	37.2	7,880	44.0	2.9	2.4	3.4	<0.001	Increasing
**Region South**	1,722	20.3	2,061	18.9	5,531	30.4	3.7	-0.2	7.7	0.065	Stationary
Paraná	556	17.7	477	11.6	1,280	19.8	1.0	-4.6	6.9	0.707	Stationary
Santa Catarina	530	24.5	600	21.5	1,584	32.7	3.2	-1.4	8.0	0.159	Stationary
Rio Grande do Sul	636	20.1	984	24.5	2,667	38.7	5.6	3.3	7.9	<0.001	Increasing
**Region Midwest**	1,369	33.9	2,113	41.7	3,690	47.4	2.6	2.1	3.0	<0.001	Increasing
Mato Grosso do Sul	166	22.4	409	40.9	476	35.2	3.0	0.8	5.2	0.011	Increasing
Mato Grosso	445	50.7	523	43.7	898	50.0	0.4	-1.3	2.2	0.630	Stationary
Goiás	734	40.0	1,164	57.0	2,105	71.1	4.2	2.5	5.9	<0.001	Increasing
Distrito Federal	24	4.1	17	2.1	211	12.6	9.2	-7.7	29.3	0.280	Stationary
**Brazil**	20,389	37.7	32,031	46.4	53,167	53.8	2.8	2.1	3.4	<0.001	Increasing

Notes: (i) absolute and relative numbers of precarious employment relationships presented for the years 2010, 2016 and 2023; (ii) time tendency analysis carried out for the period 2010-2023; *n = Absolute number; ^†^% = Percentage; ^‡^APC = Annual Percentage Change; ^§^CI95% = 95% confidence interval;^||^LL = Lower limit; ^¶^LS = Superior limit; **p-value = Probability value

The tendency in SHC was increasing in Brazil (APC = 5.1%; 95%CI = 3.1-7.0%) and in all regions of the country. The North (APC = 5.8%; 95%CI = 4.1-7.6%) and South (APC = 5.9%; 95%CI = 4.4-7.3%) regions were those that presented the greatest increases. Among the federative units, 25 (92.6%) presented an increasing tendency, one (3.7%) presented a decreasing tendency and one (3.7%) presented a stationary tendency (Table 2).

**Table 2-:** Tendency in the percentage (%) of precarious employment relationships of nurses in Secondary Health Care, based on the type of relationship, according to Brazilian states and regions, 2010-2023 (n = 8.836.941)

**Regions and Federation Units**	**Number and percentage of precarious employment relationships**									**Prais-Winsten regression**	
	**2010**		**2016**		**2023**		**APC** ^‡^	**CI95%** ^§^		*p* -value**	**Tendency**
	n*	**%** ^†^	n*	**%** ^†^	n*	**%** ^†^		**LL** ^||^	**SL** ^¶^		
**Region North**	474	26.4	2,883	43.8	2,982	56.4	5.8	4.1	7.6	<0.001	Increasing
Rondônia	31	12.8	349	22.9	309	40.8	7.3	4.9	9.7	<0.001	Increasing
Acre	7	9.6	143	15.4	128	40.1	13.1	6.6	19.9	0.001	Increasing
Amazonas	213	38.3	398	52.8	421	48.3	0.8	-3.0	4.8	0.655	Stationary
Roraima	23	39.0	119	25.2	69	25.1	-3.8	-6.3	-1.2	0.009	Decreasing
Pará	152	22.8	1,321	54.4	1,530	70.9	9.1	4.8	13.6	0.001	Increasing
Amapá	2	3.0	190	17.9	202	59.4	22.2	14.5	30.4	<0.001	Increasing
Tocantins	46	35.1	363	46.6	323	57.2	2.9	0.2	5.6	0.037	Increasing
**Region Northeast**	2,171	37.1	12,708	60.3	15,307	67.7	4.6	2.3	7.0	0.001	Increasing
Maranhão	287	46.1	1,090	70.2	1,595	69.3	2.8	0.3	5.4	0.034	Increasing
Piauí	148	39.3	801	47.7	809	58.5	2.7	1.4	4.0	0.001	Increasing
Ceará	214	29.4	2,188	57.2	2,349	72.8	7.3	4.9	9.8	<0.001	Increasing
Rio Grande do Norte	181	40.6	767	48.6	906	56.6	2.5	0.9	4.0	0.004	Increasing
Paraíba	171	39.0	1,526	64.8	1,881	66.0	3.9	0.2	7.6	0.040	Increasing
Pernambuco	353	33.4	1,931	67.1	2,222	69.9	5.2	2.2	8.3	0.003	Increasing
Alagoas	96	23.7	831	52.0	1,171	71.8	7.6	5.5	9.8	<0.001	Increasing
Sergipe	92	32.2	427	56.4	439	54.8	5.4	1.6	9.2	0.009	Increasing
Bahia	629	42.3	3,147	61.4	3,935	69.9	3.7	2.2	5.2	<0.001	Increasing
**Region Southeast**	2,956	26.5	22,136	43.2	21,221	52.3	5.1	3.0	7.4	<0.001	Increasing
Minas Gerais	824	31.3	4,986	44.3	4,067	46.8	3.4	1.2	5.8	0.006	Increasing
Espírito Santo	105	32.4	749	29.8	927	51.9	3.3	0.5	6.3	0.025	Increasing
Rio de Janeiro	734	29.3	4,966	57.5	5,500	64.6	4.3	1.6	7.2	0.005	Increasing
São Paulo	1,293	22.7	11,435	37.4	10,727	49.6	6.1	4.7	7.4	<0.001	Increasing
**Region South**	593	20.8	6,788	27.3	5,400	40.8	5.9	4.4	7.3	<0.001	Increasing
Paraná	187	19.0	2,527	22.4	1,788	35.5	5.8	2.7	8.9	0.001	Increasing
Santa Catarina	160	24.6	1,496	31.0	1,452	43.7	5.7	3.8	7.5	<0.001	Increasing
Rio Grande do Sul	246	20.2	2,765	29.8	2,160	44.2	6.4	5.7	7.1	<0.001	Increasing
**Region Midwest**	345	25.2	3,504	37.2	3,890	50.3	4.8	4.0	5.6	<0.001	Increasing
Mato Grosso do Sul	24	9.4	691	18.1	223	17.5	6.5	2.1	11.1	0.007	Increasing
Mato Grosso	134	35.6	656	39.3	965	58.4	3.5	2.0	5.0	<0.001	Increasing
Goiás	173	31.6	1,574	51.5	2,122	66.4	5.6	4.0	7.1	<0.001	Increasing
Distrito Federal	14	7.4	583	18.7	580	36.1	12.6	9.7	15.6	<0.001	Increasing
**Brazil**	6,539	28.4	48,019	45.1	48,800	54.6	5.1	3.1	7.0	<0.001	Increasing

Notes: (i) absolute and relative numbers of precarious employment relationships presented for the years 2010, 2016 and 2023; (ii) tendency time analysis carried out for the period 2010-2023; *n = Absolute number; ^†^% = Percentage; ^‡^APC = Annual Percentage Change; ^§^CI95% = 95% confidence interval;^||^LL = Lower limit; ^¶^LS = Superior limit; **p-value = Probability value

The tendency in the percentage of precarious employment relationships in the THC was increasing in Brazil (APC=6.0%; 95%CI=5.1-6.8%) and in all regions of the country, with the North region (APC=10.2%; 95%CI=7.4-13.1%) showing the highest percentage of increase. Among the federative units, 22 (81.5%) showed an increasing tendency, while five (18.5%) showed a stationary tendency (Table 3).

**Table 3-:** Tendency in the percentage (%) of precarious employment relationships for nurses in Tertiary Health Care, based on the type of relationship, according to Brazilian states and regions, 2010-2023 (n = 23.758.660)

Regions and Federation Units	Number and percentage of precarious employment relationships							Prais-Winsten regression results			
	**2010**		**2016**		**2023**		**APC** ^‡^	**CI95%** ^§^		*p* -value**	**Tendency**
	n*	**%** ^†^	n*	**%** ^†^	n*	**%** ^†^		**LL** ^||^	**SL** ^¶^		
**North Region**	503	14.7	2,562	37.7	8,610	54.0	1.2	7.4	13.1	<0.001	Increasing
Rondônia	22	7.9	140	17.3	888	39.6	11.9	7.4	16.7	<0.001	Increasing
Acre	8	2.3	56	10.4	230	28.7	17.4	8.9	26.4	0.001	Increasing
Amazonas	82	12.1	705	56.5	2,180	63.8	1.9	5.8	16.2	<0.001	Increasing
Roraima	46	28.9	33	8.7	111	10.9	-8.3	-16.0	0.1	0.055	Stationary
Pará	200	14.7	1,077	48.3	3,249	63.7	11.8	6.2	17.7	0.001	Increasing
Amapá	15	11.1	14	4.1	674	58.9	14.7	-5.9	39.8	0.159	Stationary
Tocantins	130	27.5	537	42.9	1,278	58.1	8.1	5.2	11.2	<0.001	Increasing
**Northeast Region**	3,648	24.1	11,564	42.9	34,643	56.6	6.5	4.7	8.4	<0.001	Increasing
Maranhão	778	58.5	2,085	72.5	5,105	71.2	1.3	-0.2	2.8	0.086	Stationary
Piauí	144	23.0	583	49.3	1,872	61.9	8.7	5.3	12.2	<0.001	Increasing
Ceará	327	13.5	1,089	36.7	4,839	52.4	1.8	6.9	14.9	<0.001	Increasing
Rio Grande do Norte	129	13.1	193	12.5	1,110	32.5	7.4	1.0	14.2	0.028	Increasing
Paraíba	501	36.2	1,340	53.5	2,901	63.7	4.2	2.6	5.9	<0.001	Increasing
Pernambuco	440	14.1	2,509	41.2	5,684	48.8	9.2	4.6	14.1	0.001	Increasing
Alagoas	49	12.6	309	30.1	1,387	50.5	1.4	8.1	12.9	<0.001	Increasing
Sergipe	102	20.9	243	23.4	998	39.6	6.9	2.4	11.6	0.006	Increasing
Bahia	1,178	26.9	3,213	41.4	10,747	63.8	6.6	5.3	7.8	<0.001	Increasing
**Southeast Region**	7,119	19.1	19,190	29.0	37,935	34.1	4.3	3.3	5.3	<0.001	Increasing
Minas Gerais	1,198	22.0	2,151	19.5	4,354	20.8	0.8	-1.2	2.9	0.408	Stationary
Espírito Santo	148	16.2	630	29.0	1,704	38.2	6.6	3.8	9.5	<0.001	Increasing
Rio de Janeiro	2,165	22.8	6,455	39.6	14,286	51.7	6.4	4.6	8.1	<0.001	Increasing
São Paulo	3,608	16.8	9,954	27.1	17,591	30.2	3.9	2.7	5.2	<0.001	Increasing
**South Region**	734	8.0	2,294	12,3	5,948	18.7	8.1	6.9	9.3	<0.001	Increasing
Paraná	428	13.6	1,112	16.4	2,487	21.5	5.2	2.7	7.7	0.001	Increasing
Santa Catarina	126	8.4	486	13.4	2,052	28.8	1.9	9.6	12.2	<0.001	Increasing
Rio Grande do Sul	180	4.0	696	8.4	1,409	10.8	8.6	5.0	12.4	<0.001	Increasing
**Midwest Region**	916	20.7	2,634	31.7	9,941	50.7	8.2	6.8	9.7	<0.001	Increasing
Mato Grosso do Sul	173	32.4	295	21.8	733	23.3	-2.5	-6.1	1.3	0.176	Stationary
Mato Grosso	227	32.4	549	35.2	2,427	64.5	5.5	3.4	7.7	<0.001	Increasing
Goiás	285	24.6	1,305	56.6	4,477	73.4	8.9	5.7	12.2	<0.001	Increasing
Distrito Federal	231	11.3	485	15.7	2,304	34.9	13.2	6.8	19.9	0.001	Increasing
**Brazil**	12,920	18.6	38,244	30.1	97,077	40.5	6.0	5.1	6.8	<0.001	Increasing

Notes: (i) absolute and relative numbers of precarious employment relationships presented for the years 2010, 2016 and 2023; (ii) time tendency analysis carried out for the period 2010-2023; *n = Absolute number; ^†^% = Percentage; ^‡^APC = Annual Percentage Change; ^§^CI95% = 95% confidence interval; ^||^LL = Lower limit; ^¶^SL = Superior Limit; **p-value = Probability value

**Figure 2-:**
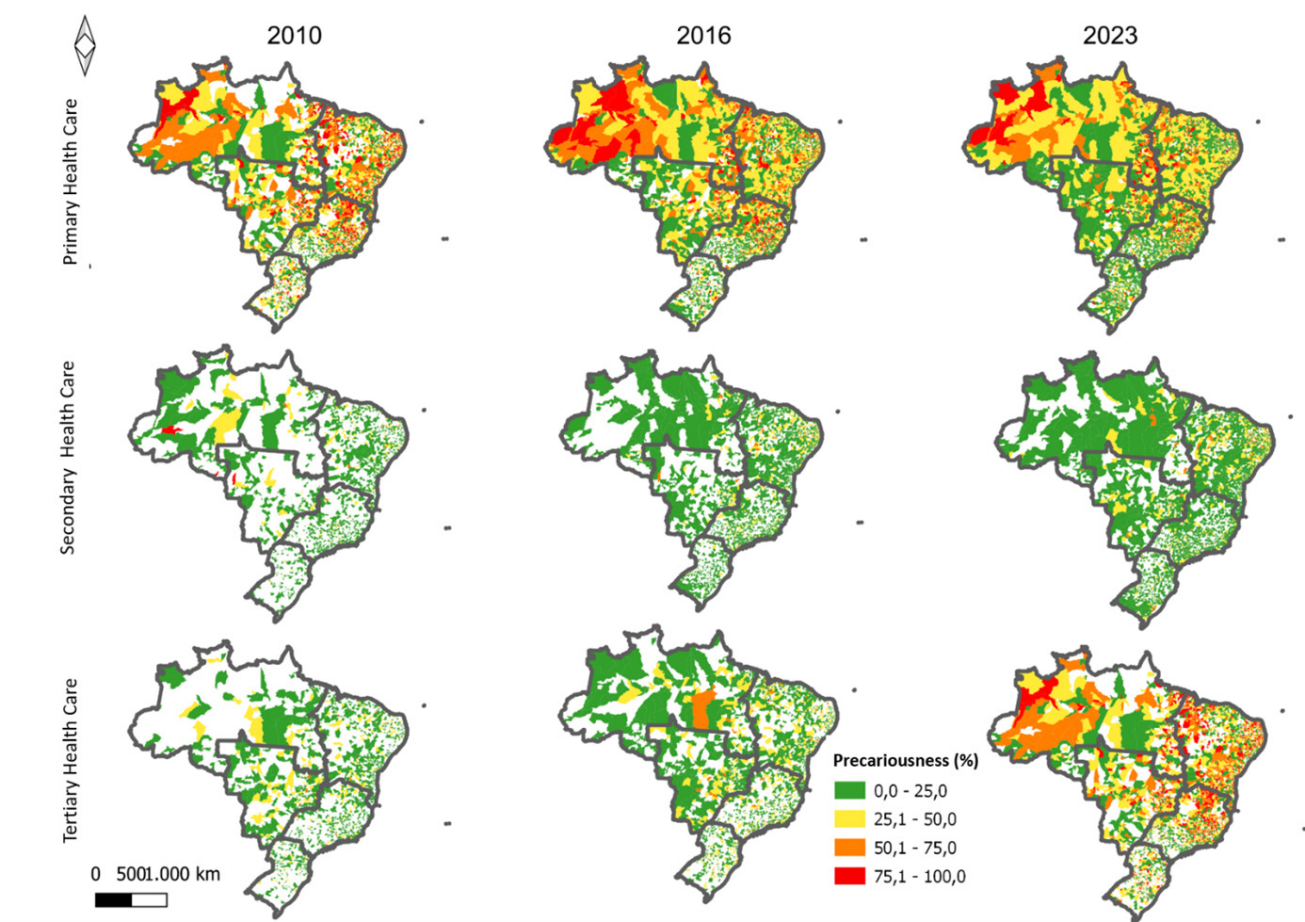
Spatial distribution of the percentage (%) of precarious employment relationships for nurses, based on the type of relationship, according to the level of health care in Brazilian municipalities, 2010, 2016 and 2023

Notes: (i) white municipalities indicate missing data for professional ties in the NRHE and (ii) number of observations (n) included in the spatial distribution analysis: (ii.1): Primary Health Care: 105,587 (years 2010, 2016 and 2023); (ii.2): Secondary Health Care: 103,358 (years 2010, 2016 and 2023); (ii.3): Tertiary Health Care: 148,241 (years 2010, 2016 and 2023)

Figure 2 shows the spatial distribution of the percentage of nurses with precarious employment contracts in Brazilian municipalities in 2010, 2016 and 2023, according to the level of health care. It is possible to observe an increase in the number of municipalities with a high percentage of precarious employment contracts in the period. This increase is more visible for THC. It is also observed that the North and Northeast regions, in 2023, had the highest percentages of precarious employment contracts for nurses in PHC and THC.

The results showed an increase in the precariousness of nursing work in Brazil based on the type of employment relationship and the need for strategies to reduce precariousness in the SUS.

## Discussion

The results of this study indicated an increasing tendency in nurses’ PER at all levels of health care in Brazil, with the greatest increases in SHC and THC. For PHC, the increasing tendency in PER occurred in all regions except the South. In SHC and THC, there was an increase in PER in all regions of the country. Regardless of the level of care, most federative units showed an increasing tendency. In 2023, PHC and SHC had the highest percentages of PW bonds when compared to THC. The spatial analysis showed an increase in the number of municipalities with a high percentage of PW bonds for nurses, especially in THC.

Most studies that analyzed PER focused on analyzing the type of bond as a proxy for the PW indicator^(6,30-32)^, an approach similar to that of this study. In general, studies have associated some employment relationships with PW, since they had low income levels, reduced rights and legal protection for employment, including: self-employed professionals directly or through intermediaries from civil society organizations, philanthropic or non-profit entities, cooperatives, private companies, non-governmental companies and social organizations, as well as scholarship holders, professionals in commissioned positions, consultants and those with fixed-term contracts^(30-32)^. Other approaches, however, have considered multidimensional aspects to measure the precariousness of employment relationships, including the use of broad scales^(32-35)^.

No tendency studies were found with the same methodological approach for comparison with this investigation. However, the results were similar to a study conducted with data from 2007 to 2021 that showed an increase in the percentage of PW contracts in physical education professionals working in the SUS, which identified that the PHC had the highest number of registrations of professionals with precarious employment contracts, the opposite of what occurs in the SHC and THC^(6)^. Another investigation that analyzed data from the Program for Improving Access to Quality in Primary Care showed a high proportion of nurses with PW contracts, such as temporary contracts or contracts brokered by Social Organizations, in addition to a tendency of retraction of stable contracts in the PHC^(11)^. The results of this study and previous evidence suggest that PER in the PHC is an emerging and growing problem in Brazil.

The growing scenario of PER among nurses in PHC in the country is a concern. PHC acts as a guiding axis of care and a priority model for organizing the SUS. It is characterized by individual and collective actions that include health promotion and protection, disease prevention, diagnosis, treatment, rehabilitation, and health maintenance^(36)^. The direct impact of PER on PHC is the high turnover of nurses, generating work overload for other professionals, compromising the establishment of bonds with the population served, and reducing the quality of care^(11,30)^.

In summary, the present study showed a higher percentage of increase in PW bonds in SHC and THC when compared to PHC. No tendency studies were found that compared percentage variations according to the level of care. However, the study that analyzed PER in physical education professionals showed an increase in the number of registrations of professionals with precarious bonds in SHC and THC when compared to PHC^(6)^. Some hypotheses for this sharper increase in SHC and THC may include later public-private participation in these levels of care, including the increase in contracts with Social Organizations in specialized care^(6)^. In addition, as of 2020, the pandemic of the disease caused by the new coronavirus (COVID-19) required new hiring by managers, given the overload of health services, especially in specialized care^(2)^. This aspect may have contributed to the greater increase in PER of nurses in specialized care when compared to PHC^(15)^.

The highest percentage of PW bonds of nurses was found in the North and Northeast regions. This result was also observed in physical education professionals^(6)^. Studies show that the North and Northeast regions concentrate the smallest municipalities in the country and poorer health infrastructure conditions when compared to other regions^(22-23)^. These factors, among others, generate greater difficulty in attracting and retaining qualified health professionals^(37-38)^ and contribute to the greater search for precarious forms of hiring in these regions^(6)^. Some indicators may point to possible impacts of PER on the retention of professionals and health care in these locations. For example, the North and Northeast regions have the lowest ratio of health professionals per inhabitant and lower percentages of access to health services compared to other regions^(39-40)^.

The high percentage of PW contracts at all levels of health care and its increasing tendency still persists as a problem for the health system, even with the policies and initiatives to combat it implemented by the Ministry of Health, such as *DesprecarizaSUS* ^(19)^. The tendency for the percentage of precarious work contracts to increase may be related to a set of factors, such as the problems of underfunding of the SUS^(12)^.

From an operational point of view, precariousness practices aim to allow flexibility and reduce costs for health services^(41)^. However, some studies have shown that the high percentage of PER affects the organization of health service management, reduces the expansion of coverage of actions and services and the quality and comprehensiveness of care and may impact on negative outcomes for users and reduced access to services^(2,42)^. In addition, the commodification and disposability of HWF, as well as the change in management standards and work organization caused by PER, also lead to vulnerability, increased risks related to safety and health conditions at work, exposing professionals to various physical and mental health problems. Furthermore, the isolation resulting from this practice, in addition to the devaluation arising from it, may negatively affect class solidarity, leading to the cooling of union strength^(43)^.

This study had limitations, such as the quality of the data available in the NRHE, which may lead to underestimation or overestimation of the indicator of the percentage of precarious employment relationships. On the other hand, the database is reliable^(44)^ and is frequently used in studies in the area. The study focused on investigating PER, not covering other dimensions of precarious work in health, such as multiple relationships, low pay, lack of work and rest infrastructure, violence, harassment, repetitive strain, and the consequences of PER for the health of workers and the quality of care for users^(2,41)^. The database also did not allow us to assess the number of relationships of the same nurse, due to the lack of identification of the participants. Therefore, it was not possible to analyze the temporal tendency of double or triple relationships of these professionals. Finally, the present investigation included data before and during the COVID-19 pandemic, a period in which many nurses were hired to meet emergency health demands, mostly through temporary contracts and, at times, without guarantees of social rights. This fact may contribute to reducing the accuracy in determining the temporal tendency.

However, this study presented important advances in the knowledge of nurses’ PER, exploring temporal trends of this dimension disaggregated by regions, federation units and level of health care, supporting monitoring and the need for public policies that promote the de-precariousness of work in the SUS.

New research can analyse the determinants of nurses’ PER, such as the analysis of variables of a socioeconomic nature [for example, Gross Domestic Product (GDP) *per capita*, average salary, *per capita* health expenditure, etc.] and infrastructure (such as types of units), among others, contributing to understanding the PER phenomenon, especially in certain regions and states of Brazil. In addition to these determinants, it is important to understand how precariousness affects some outcomes, whether in the efficiency of health systems and services or in the quality of care. Understanding the precariousness of HWF requires a multidimensional approach. In addition to the types of employment relationships analyzed in this study, future research could incorporate other secondary sources to examine additional components, such as average income, multiplicity of employment relationships, and illness among workers, enabling the construction of a multidimensional index of precariousness. There is a wealth of evidence showing adverse effects of precariousness on the health of health workers at the individual level, such as illness and socioeconomic vulnerability. However, it is important to investigate, at the ecological level, how precariousness can influence elements such as retention, density of professionals, and quality of health care.

## Conclusion

This study showed an increasing trend for PER among nurses in Brazil, regardless of the level of care and geographic region, based on the type of employment relationship. The North and Northeast regions had the highest percentages of PW employment relationships, suggesting inequalities in PER across the country. The results showed an increase in the precariousness of nurses’ employment relationships and the need for strategies to reduce precariousness in the SUS.

## Data Availability

The dataset of this article is available at https://drive.google.com/drive/folders/1rEtK9rJJKjRWVFd6OByr_MPx8m9cxkHe

## Footnotes

1 SUS’ acronym is well-known in Brazil and stands for “*Sistema Único de Saúde*”, in Portuguese or Unified Health System, in English. Therefore, we will keep its original acronym throughout the paper.
