# Supplementary material for: Trends and spatial distribution of precarious work conditions for nurses in Brazil based on the type of employment bond
Source: Rev Lat Am Enfermagem. 2025 Nov 17;33:e4644. doi: 10.1590/1518-8345.7680.4644 (PMC12628371; doi:10.1590/1518-8345.7680.4644)
Supplement: Translation

# Translation: Tendências e distribuição espacial da precarização do trabalho dos enfermeiros no Brasil com base no tipo de vínculo*

**DOI:** 10.1590/1518-8345.7680.4645

**Authors:** Érika Carvalho de Aquino, Rafael Alves Guimarães, Daniel do Prado Pagotto, Joseane Aparecida Duarte, Antônio Isidro da Silva, Cândido Vieira Borges

**Affiliations:** 1 Universidade Federal de Goiás, Instituto de Patologia Tropical e Saúde Pública, Goiânia, GO, Brasil.; 2 Universidade Federal de Goiás, Faculdade de Enfermagem, Goiânia, GO, Brasil.; 3 Universidade de Brasília, Brasília, DF, Brasil.; 4 Universidade Federal da Bahia, Salvador, BA, Brasil.; 5 Universidade Federal de Goiás, Faculdade de Administração, Ciências Contábeis e Ciências Econômicas, Goiânia, GO, Brasil.

**Keywords:** Força de Trabalho, Condições de Trabalho, Trabalho Precário, Segurança do Emprego, Enfermeiras e Enfermeiros, Níveis de Atenção à Saúde

## Abstract

analisar a tendência e a distribuição espacial da precarização do trabalho dos enfermeiros no Brasil, com base no tipo de vínculo, entre 2010 e 2023, de acordo com o nível de atenção à saúde.

estudo ecológico com dados extraídos do Cadastro Nacional de Estabelecimentos de Saúde. O indicador analisado foi o percentual de vínculos de trabalho precário. A tendência temporal foi avaliada pelo modelo de regressão de Prais-Winsten e a distribuição espacial, por meio de mapas coropléticos.

houve uma tendência crescente da precarização do trabalho dos enfermeiros no Brasil, com base no tipo de vínculo, independentemente do nível de atenção à saúde. A região Norte apresentou os maiores percentuais de aumento do indicador na Atenção Primária à Saúde, Atenção Secundária à Saúde e Atenção Terciária à Saúde. Já as regiões Norte e Nordeste concentraram os maiores números de municípios com alto percentual de vínculos de trabalho precário.

a precarização dos vínculos de trabalho em enfermagem, com base no tipo de vínculo, apresentou tendência crescente em todos os níveis de atenção à saúde, sendo mais elevada na Atenção Primária à Saúde e com maior incremento na Atenção Terciária à Saúde, dentro do horizonte temporal analisado.

## Destaques

**(1)** A precarização dos vínculos de trabalho de enfermeiros aumentou substancialmente. **(2)** A precarização dos vínculos foi alta na Atenção Primária à Saúde. **(3)** As regiões Norte e Nordeste apresentaram maior precarização dos vínculos. **(4)** A Atenção Terciária à Saúde registrou maior incremento na precarização dos vínculos.

## Introdução

A Precarização dos Vínculos de Trabalho (PVT) representa um grave problema global e tem consequências adversas na saúde dos trabalhadores, na gestão e na qualidade da assistência em saúde^(1-2)^, diminuindo a capacidade dos sistemas de saúde em atingir os seus objetivos, sobretudo a Cobertura Universal de Saúde^(3)^. O Trabalho Precário (TP) é uma construção multidimensional caracterizado por condições de emprego de baixa qualidade (incluindo a curta duração do vínculo empregatício), vínculos temporários, baixa remuneração, falta de direitos e proteção aos trabalhadores, além de insegurança laboral^(4-5)^.

No Brasil, a PVT representa um desafio para o Sistema Único de Saúde (SUS) com tendência crescente^(6)^, sendo potencializada por políticas neoliberais, bem como pelo aumento da terceirização e da flexibilização dos contratos^(7)^ em decorrência de mudanças legislativas e restrições orçamentárias do sistema de saúde, tais quais as Emendas Constitucionais número 19 de 1998^(8)^ e número 95 de 2016^(9)^, além do estabelecimento de parcerias públicas e privadas para a constituição de Redes de Atenção à Saúde^(7)^. Essas medidas agravaram as desigualdades regionais e prejudicaram a alocação de profissionais qualificados nos territórios^(10)^. Outros estudos mostram que a flexibilização dos contratos, com destaque para a terceirização e a quarteirização, resultou em vínculos trabalhistas mais precários, comprometendo a qualidade dos serviços prestados no SUS^(11-12)^.

Uma das categorias profissionais que atuam no SUS e que mais foram impactadas pela PVT é a enfermagem, composta de enfermeiros, técnicos e auxiliares em enfermagem^(2-13)^. A enfermagem compõe a maior Força de Trabalho em Saúde (FTS) do país^(14)^, apresentando um papel fundamental no desempenho do SUS em todos os níveis de atenção. Apesar disso, muitos profissionais de enfermagem enfrentam condições de trabalho precárias, como vínculos temporários e sem garantias de direitos sociais, baixa remuneração e sobrecarga^(15-16)^. Essa realidade compromete a capacidade do sistema em oferecer serviços de saúde de qualidade e em reter profissionais em regiões de baixa densidade populacional^(16)^.

Apesar disso, nenhum estudo nacional amplo com dados populacionais foi realizado para avaliar as tendências da PVT de enfermeiros, sobretudo desagregado por regiões e unidades da federação. Além disso, esse fenômeno pode se comportar de forma distinta entre os níveis de atenção à saúde, com a Atenção Primária à Saúde (APS) englobando a maior parte dos vínculos precários^(6)^. Portanto, há também uma lacuna na literatura sobre as tendências da PVT de enfermeiros segundo o nível de atenção à saúde. Diante deste cenário, o estudo das tendências e da distribuição espacial da PVT para enfermeiros mostra-se fundamental e emergente no país, uma vez que está alinhado às agendas internacionais, como o oitavo Objetivo de Desenvolvimento Sustentável da Organização das Nações Unidas^(17)^ e a Estratégia Global de Recursos Humanos em Saúde da Organização Mundial da Saúde^(18)^, além de contribuir para subsidiar estratégias de desprecarização dos vínculos de trabalho na saúde, como o Programa Nacional de Desprecarização do Trabalho no SUS^(19)^.

Dessa forma, o objetivo deste estudo foi analisar a tendência e a distribuição espacial da precarização do trabalho dos enfermeiros no Brasil, com base no tipo de vínculo, entre 2010 e 2023, de acordo com o nível de atenção à saúde.

## Método

### Desenho do estudo

Estudo ecológico de séries temporais e do tipo base populacional. O relato desse manuscrito foi realizado utilizando adaptações do *Revised Standards for Quality Improvement Reporting Excellence* (SQUIRE 2.0)^(20)^.

### Contexto

O estudo foi conduzido no Brasil, com base nos dados de todos os municípios brasileiros, de 2010 a 2023. Esse período foi escolhido por permitir a análise de uma linha histórica mais ampla, fornecendo subsídios para a discussão de diversas legislações, programas e políticas públicas que contribuíram para a PVT nesse intervalo. Além disso, esse recorte temporal incluiu tanto o período da pandemia de COVID-19 quanto uma época anterior a ela, possibilitando a avaliação de mudanças no contexto de vínculos profissionais durante e após a crise sanitária, na qual muitos enfermeiros foram submetidos aos vínculos de trabalho precários^(12)^.

Vale ressaltar que o país apresentava uma população estimada em 203.080.756 milhões de habitantes em 2022, conforme o último censo demográfico do Instituto Brasileiro de Geografia e Estatística, distribuídos em 5.568 municípios^(21)^, agrupados em 26 unidades da federação e o Distrito Federal. Ademais, as regiões apresentam distintas características demográficas, sociais e econômicas, além de diferenças do contingente de enfermeiros e do número de dispositivos e investimentos nas Redes de Atenção à Saúde^(22-23)^.

### Participantes

Foram incluídos no estudo todos os vínculos de trabalho de enfermeiros que atuavam em serviços de saúde no Brasil e que estavam cadastrados no Cadastro Nacional de Estabelecimentos de Saúde (CNES), entre 2010 e 2023, independentemente do nível de atuação (primário, secundário e terciário).

### Fonte de dados e variáveis

Os dados dos vínculos de trabalhos de enfermeiros atuantes nos serviços de saúde no Brasil foram extraídos de microdados do CNES, no *layout* dos arquivos de profissionais, disponível de modo público. A extração foi realizada por meio do protocolo de transferência de arquivos do Departamento de Informática do SUS, em 10 de janeiro de 2024.

O CNES é um Sistema de Informação em Saúde que contém dados sobre a estrutura física das unidades de saúde, os serviços de saúde disponíveis e os profissionais vinculados aos estabelecimentos de saúde do território nacional, em todas as esferas de gestão (nacional, estadual e municipal), vinculados ou não ao SUS. Ademais, os dados são inseridos por meio do preenchimento de formulários com variáveis sobre a estrutura física, os equipamentos, FTS, dentre outros elementos^(24)^.

Assim, os seguintes dados foram extraídos do CNES: o código do município do estabelecimento de saúde, código CNES da unidade, o tipo de unidade, o código brasileiro de ocupações (CBO), o tipo de vínculo do profissional e o subtipo de vínculo do profissional. Também foram filtrados os CBO cuja família de CBO era iniciada pelo código 2235, correspondente aos enfermeiros e afins.

Ainda, vale ressaltar que os microdados são a menor desagregação do CNES, significando que cada uma das observações representa um vínculo laboral. Esses vínculos foram organizados de acordo com o tipo e subtipo de vínculo, extraído do campo “vínculo empregatício com o estabelecimento”. De acordo com um estudo anterior^(6)^, os vínculos foram classificados como: (i) protegidos: servidor público, servidor público cedido para iniciativa privada, celetista (empregado regido pela legislação brasileira CLT - Consolidação das Leis do Trabalho), empregado público e estatutário; (ii) precários: autônomos (incluídos aqueles com vínculo direto, sem intermediação ou aqueles com vínculo indireto, quando intermediado por instituições ou entidades como cooperativas, Organizações da Sociedade Civil de Interesse Público, entidade filantrópica e/ou sem fins lucrativos, empresa privada, Organizações Não Governamentais e Organizações Sociais), bolsistas, cooperados, contratos verbais ou informais, voluntariados, cargos comissionados, contratos temporários ou por prazo determinado; (iii) outros: proprietários, estagiários e residentes e (iv) sem informação: tipo de vínculo ausente no CNES.

Desse modo, os vínculos laborais foram classificados de acordo com o nível de atenção em: (i) APS^(6,25)^, que incluiu os seguintes códigos do CNES: 01 – posto de saúde, 02 – centro de saúde/unidade básica, 32 – unidade móvel fluvial, 40 – unidade móvel terrestre, 71 – centro de apoio à saúde da família, 72 – unidade de atenção à saúde indígena e 74 – polo academia da saúde; (ii) Atenção Secundária à Saúde (ASS), que englobou os seguintes códigos: 04 – policlínica, 15 – unidade mista, 20 – pronto socorro geral, 21 – pronto socorro especializado, 22 – consultório isolado, 36 – clínica/centro especializado, 39 – unidade de apoio diagnóstico e terapia, 42 – unidade móvel de nível pré-hospitalar na área de urgência, 61 – centro de parto normal – isolado, 62 – hospital/dia-isolado, 69 – centro de atenção hemoterapia e/ou hematológica, 70 – CAPS, 73 – pronto atendimento e 83 – polos de prevenção de doenças e agravos e promoção da saúde e (iii) Atenção Terciária à Saúde (ATS), que incluiu os códigos: 05 – hospital geral e 07 – hospital especializado. Assim, foram excluídos vínculos associados aos demais estabelecimentos de saúde.

Similarmente, estudos anteriores também empregaram análises sobre os tipos de vínculos de trabalho para investigar a precarização^(6,11,25)^. No entanto, a classificação adotada neste estudo foi escolhida por ser categórica em relação aos tipos de vínculo e seus respectivos graus de precarização^(6)^, além de ser compatível com a estrutura de dados utilizada, o CNES.

Além do mais, o indicador analisado foi o percentual (%) de vínculos de trabalho precário de enfermeiros. Esse indicador foi calculado, para cada ano, por meio da seguinte fórmula:

$$\frac{Númerodevínculoslaboralesprecariosendeterminadalocalidadyperíodo}{Númerototaldevínculosnamesmalocalidadeperíodo}x100$$

O horizonte temporal foi de 2010 a 2023. Foram analisados os números de vínculos referentes ao mês de junho de cada ano, por tratar-se do meio do ano, além de haver menos influência de mudanças de gestão nesse período e ser congruente com indicadores populacionais anuais que também fazem referência a esse mês.

### Análise estatística

Inicialmente, foram realizadas análises descritivas por meio do número absoluto da PVT e do percentual (%) de vínculos de trabalho precário.

A seguir, foram analisadas as tendências temporais pelo modelo de regressão linear de Prais-Winsten. Antes da inclusão nos modelos de regressão, a transformação logarítmica de base 10 das taxas foi realizada para reduzir a heterogeneidade da variância dos resíduos e contribuir para a apuração da tendência temporal^(26)^. Ademais, a variável dependente (Y) utilizada foi o percentual (%) de vínculos de trabalho precário, enquanto a variável independente (X) foi o ano da série temporal. Logo, a equação de regressão foi definida por ^(26)^, sendo os percentuais (%) de vínculos de trabalho precário de enfermeiros após a transformação logarítmica, β_0_ o intercepto ou constante da regressão, β_1_o coeficiente de inclinação da reta e *e_t_* o erro aleatório. O “*t*” estima os tempos do conjunto de dados {*t*_1_, ..., *t*_14_}, no caso *t*_1_=2010 e *t*_14_=2023.

Por meio dos modelos de regressão, foi possível obter o valor do coeficiente de inclinação da reta (β_1_) e os erros padrões (EP). Com esses parâmetros, calculou-se a Variação Percentual Anual (VPA), de acordo com a seguinte fórmula^(26)^:

$$VPA=\left(1+10^{\beta_{1}}\right)*100$$

sendo β_1_ o coeficiente de inclinação da reta.

Os limites inferiores e superiores do Intervalo de Confiança de 95% (IC95%) da VPA foram calculados por meio da fórmula^(26)^:

$$IC95\%=\left(1+10^{\beta_{1}\left(t*EP\right)}\right)*100$$

sendo β_1_ o coeficiente de inclinação da reta obtida no modelo de regressão, *t* o valor que a distribuição t de Student apresenta 13 graus de liberdade (*n*-1) a um IC95% bicaudal e EP o erro padrão da estimativa de β_1_obtido na regressão.

As análises foram realizadas de forma desagregada, segundo o nível de atenção: (i) APS, (ii) ASS e (iii) ATS para o Brasil, as cinco grandes regiões e as 27 unidades da federação, constituindo 99 séries temporais analisadas. Além disso, foram realizadas análises da distribuição espacial do indicador, segundo o nível de atenção para os anos de 2010, 2016 e 2023. Também, foram construídos mapas coropléticos, utilizando como unidade de análise o número de municípios brasileiros (n=5.568).

Por fim, as extrações e tratamentos dos dados foram realizados usando a linguagem *Structured Query Language* (SQL). As análises descritivas e os modelos de séries temporais foram realizados utilizando a linguagem R, com interface RStudio^(27)^. As análises de distribuição espacial foram conduzidas com o uso do *software* QGIS (QGIS Development Team, 2024)^(28)^.

### Aspectos éticos

Não houve necessidade de submissão ao Comitê de Ética em Pesquisa, uma vez que foram utilizados exclusivamente microdados secundários e de acesso público, por meio da ferramenta de transferência de arquivos do DATASUS, conforme dispensa prevista na Resolução nº 510/2016 do Conselho Nacional de Saúde^(29)^.

## Resultados

Entre 2010 e 2023, foram registrados 47.411.575 vínculos de trabalhos de enfermeiros no CNES. Destes, foram excluídos 2.629.306 (5,54%) registros relacionados às unidades de saúde cujos tipos não fizeram parte da análise. Dessa forma, foram analisados 44.782.269 vínculos laborais, representando 94,46% do total dos vínculos no período. Desses, 12.186.668 (27,21%) eram vínculos em unidades da APS, 8.836.941 (19,73%) da ASS e 23.758.660 (53,05%) da ATS.

A Figura 1 apresenta a evolução do percentual (%) de vínculos de trabalho precário de enfermeiros, conforme nível de atenção e regiões. No Brasil, o percentual de vínculos precários foi semelhante na APS e ASS e menor nas ATS. Esse padrão é similar nas regiões Sudeste e Nordeste. Todavia, a região Sul apresenta o maior percentual dos vínculos precários na ASS, quando comparado aos demais níveis de atenção, enquanto a região Norte apresenta o maior percentual na APS. Na região Centro-Oeste, os percentuais de vínculos precários entre os níveis de atenção apresentam aproximação ao longo do tempo, com percentuais similares a partir de 2021.

Como resultado, o Brasil apresentou uma tendência crescente do percentual de PVT na APS (VPA = 2,8%; IC95%=2,1-3,4%). Todas as regiões apresentaram tendência crescente do indicador na APS, exceto a região Sul. A região Norte foi a que apresentou o maior percentual de incremento (VPA=4,8%; IC95%=3,1-6,6%). Entre as unidades da federação, 21 (77,8%) apresentaram tendência crescente, enquanto seis (22,2%) apresentaram tendência estacionária do percentual de PVT de enfermeiros na APS (Tabela 1).

**Figura 1-:** Percentual (%) de vínculos de trabalhos precários de enfermeiros, segundo nível de atenção à saúde e regiões do Brasil, 2010-2023 (n = 44.782.269)

Nota: Número de observações (n) incluídas na análise das séries históricas: (ii.1): Atenção Primária à Saúde: 12.186.668; (ii.2): Atenção Secundária à Saúde: 8.836.941 e (ii.3): Atenção Terciária à Saúde: 23.758.660.

**Tabela 1-:** Tendência do percentual (%) de vínculos de trabalho precário de enfermeiros na Atenção Primária à Saúde, com base no tipo de vínculo, segundo unidades da federação e regiões do Brasil, 2010-2023 (n = 12.186.668)

**Regiões e Unidades da Federação**	**Número e percentual de vínculos de trabalho precário**							**Regressão de Prais-Winsten**			
	**2010**		**2016**		**2023**		**VPA** ^‡^	**IC95%** ^§^		Valor de *p* **	**Tendência**
	n*	**%** ^†^	n*	**%** ^†^	n*	**%** ^†^		**LI** ^||^	**LS** ^¶^		
**Região Norte**	1.758	39,1	3.717	59,3	6.468	72,4	4,8	3,1	6,6	<0,001	Crescente
Rondônia	65	14,2	108	17,9	264	33,5	7,6	4,8	10,5	<0,001	Crescente
Acre	58	20,8	124	32,7	211	43,7	6,1	3,6	8,7	<0,001	Crescente
Amazonas	506	53,4	976	72,1	1.730	85,9	3,7	2,8	4,6	<0,001	Crescente
Roraima	96	61,1	152	56,3	267	60,7	0,1	-3,1	3,4	0,947	Estacionária
Pará	733	39,6	1.746	67,8	2.744	78,2	5,3	2,7	8,0	0,001	Crescente
Amapá	9	3,6	182	56,0	419	74,3	25,5	7,0	47,1	0,010	Crescente
Tocantins	291	52,7	429	56,0	833	73,9	3,6	2,7	4,5	<0,001	Crescente
**Região Nordeste**	7.865	45,4	11.689	57,5	18.128	67,6	3,1	2,4	3,8	<0,001	Crescente
Maranhão	1.376	60,9	1.991	73,4	2.850	82,9	2,3	1,6	3,0	<0,001	Crescente
Piauí	258	21,4	555	36,2	983	49,1	7,0	5,3	8,7	<0,001	Crescente
Ceará	739	27,3	1.684	51,5	2.793	62,4	6,5	4,1	8,9	<0,001	Crescente
Rio Grande do Norte	685	62,4	754	57,9	1.033	63,1	0,5	-0,1	1,0	0,101	Estacionária
Paraíba	739	46,6	883	49,9	1.347	59,3	2,3	1,5	3,1	<0,001	Crescente
Pernambuco	1.416	60,0	1.997	69,5	2.560	70,7	1,2	0,3	2,0	0,013	Crescente
Alagoas	240	24,5	391	34,5	997	60,7	7,1	5,4	8,7	<0,001	Crescente
Sergipe	130	18,4	247	31,9	775	63,3	11,1	10,2	12,1	<0,001	Crescente
Bahia	2.282	51,8	3.187	64,1	4.790	73,6	2,6	1,6	3,5	<0,001	Crescente
**Região Sudeste**	7.675	38,7	12.451	47,1	19.350	52,1	2,1	1,4	2,8	<0,001	Crescente
Minas Gerais	2.857	44,9	4.408	53,2	6.383	55,0	1,5	0,6	2,3	0,004	Crescente
Espírito Santo	496	41,8	565	47,7	1.207	65,5	3,3	1,6	5,0	0,001	Crescente
Rio de Janeiro	1.540	50,3	2.870	62,6	3.880	67,2	1,9	0,8	3,0	0,003	Crescente
São Paulo	2.782	30,2	4.608	37,2	7.880	44,0	2,9	2,4	3,4	<0,001	Crescente
**Região Sul**	1.722	20,3	2.061	18,9	5.531	30,4	3,7	-0,2	7,7	0,065	Estacionária
Paraná	556	17,7	477	11,6	1.280	19,8	1,0	-4,6	6,9	0,707	Estacionária
Santa Catarina	530	24,5	600	21,5	1.584	32,7	3,2	-1,4	8,0	0,159	Estacionária
Rio Grande do Sul	636	20,1	984	24,5	2.667	38,7	5,6	3,3	7,9	<0,001	Crescente
**Região Centro-Oeste**	1.369	33,9	2.113	41,7	3.690	47,4	2,6	2,1	3,0	<0,001	Crescente
Mato Grosso do Sul	166	22,4	409	40,9	476	35,2	3,0	0,8	5,2	0,011	Crescente
Mato Grosso	445	50,7	523	43,7	898	50,0	0,4	-1,3	2,2	0,630	Estacionária
Goiás	734	40,0	1.164	57,0	2.105	71,1	4,2	2,5	5,9	<0,001	Crescente
Distrito Federal	24	4,1	17	2,1	211	12,6	9,2	-7,7	29,3	0,280	Estacionária
**Brasil**	20.389	37,7	32.031	46,4	53.167	53,8	2,8	2,1	3,4	<0,001	Crescente

Notas: (i) números absolutos e relativos de vínculos de trabalhos precários apresentados para os anos de 2010, 2016 e 2023; (ii) análise de tendência temporal realizada para o período 2010-2023; *n = Número absoluto; ^†^% = Percentual; ^‡^VPA = Variação percentual anual; ^§^IC95% = Intervalo de confiança de 95%; ^||^LI =Limite inferior; ^¶^LS = Limite superior; **Valor de *p* = Valor de probabilidade

A tendência na ASS foi crescente no Brasil (VPA = 5,1%; IC95%=3,1-7,0%), assim como em todas as regiões do país. As regiões Norte (VPA=5,8%; IC95%=4,1-7,6%) e Sul (VPA=5,9%; IC95%=4,4-7,3%) foram as que apresentaram os maiores aumentos. Entre as unidades da federação, 25 (92,6%) apresentaram tendência crescente, uma (3,7%) apresentou tendência decrescente e uma (3,7%) apresentou tendência estacionária (Tabela 2).

**Tabela 2-:** Tendência do percentual (%) de vínculos trabalho precário de enfermeiros na Atenção Secundária à Saúde, com base no tipo de vínculo, segundo unidades da federação e regiões do Brasil, 2010-2023 (n = 8.836.941)

**Regiões e Unidades da Federação**	**Número e percentual de vínculos de trabalho precário**							**Regressão de Prais-Winsten**			
	**2010**		**2016**		**2023**		**VPA** ^‡^	**IC95%** ^§^		Valor de *p* **	**Tendência**
	n*	**%** ^†^	n*	**%** ^†^	n*	**%** ^†^		**LI** ^||^	**LS** ^¶^		
**Região Norte**	474	26,4	2.883	43,8	2.982	56,4	5,8	4,1	7,6	<0,001	Crescente
Rondônia	31	12,8	349	22,9	309	40,8	7,3	4,9	9,7	<0,001	Crescente
Acre	7	9,6	143	15,4	128	40,1	13,1	6,6	19,9	0,001	Crescente
Amazonas	213	38,3	398	52,8	421	48,3	0,8	-3,0	4,8	0,655	Estacionária
Roraima	23	39,0	119	25,2	69	25,1	-3,8	-6,3	-1,2	0,009	Decrescente
Pará	152	22,8	1.321	54,4	1.530	70,9	9,1	4,8	13,6	0,001	Crescente
Amapá	2	3,0	190	17,9	202	59,4	22,2	14,5	30,4	<0,001	Crescente
Tocantins	46	35,1	363	46,6	323	57,2	2,9	0,2	5,6	0,037	Crescente
**Região Nordeste**	2.171	37,1	12.708	60,3	15.307	67,7	4,6	2,3	7,0	0,001	Crescente
Maranhão	287	46,1	1.090	70,2	1.595	69,3	2,8	0,3	5,4	0,034	Crescente
Piauí	148	39,3	801	47,7	809	58,5	2,7	1,4	4,0	0,001	Crescente
Ceará	214	29,4	2.188	57,2	2.349	72,8	7,3	4,9	9,8	<0,001	Crescente
Rio Grande do Norte	181	40,6	767	48,6	906	56,6	2,5	0,9	4,0	0,004	Crescente
Paraíba	171	39,0	1.526	64,8	1.881	66,0	3,9	0,2	7,6	0,040	Crescente
Pernambuco	353	33,4	1.931	67,1	2.222	69,9	5,2	2,2	8,3	0,003	Crescente
Alagoas	96	23,7	831	52,0	1.171	71,8	7,6	5,5	9,8	<0,001	Crescente
Sergipe	92	32,2	427	56,4	439	54,8	5,4	1,6	9,2	0,009	Crescente
Bahia	629	42,3	3.147	61,4	3.935	69,9	3,7	2,2	5,2	<0,001	Crescente
**Região Sudeste**	2.956	26,5	22.136	43,2	21.221	52,3	5,1	3,0	7,4	<0,001	Crescente
Minas Gerais	824	31,3	4.986	44,3	4.067	46,8	3,4	1,2	5,8	0,006	Crescente
Espírito Santo	105	32,4	749	29,8	927	51,9	3,3	0,5	6,3	0,025	Crescente
Rio de Janeiro	734	29,3	4.966	57,5	5.500	64,6	4,3	1,6	7,2	0,005	Crescente
São Paulo	1.293	22,7	11.435	37,4	10.727	49,6	6,1	4,7	7,4	<0,001	Crescente
**Região Sul**	593	20,8	6.788	27,3	5.400	40,8	5,9	4,4	7,3	<0,001	Crescente
Paraná	187	19,0	2.527	22,4	1.788	35,5	5,8	2,7	8,9	0,001	Crescente
Santa Catarina	160	24,6	1.496	31,0	1.452	43,7	5,7	3,8	7,5	<0,001	Crescente
Rio Grande do Sul	246	20,2	2.765	29,8	2.160	44,2	6,4	5,7	7,1	<0,001	Crescente
**Região Centro-Oeste**	345	25,2	3.504	37,2	3.890	50,3	4,8	4,0	5,6	<0,001	Crescente
Mato Grosso do Sul	24	9,4	691	18,1	223	17,5	6,5	2,1	11,1	0,007	Crescente
Mato Grosso	134	35,6	656	39,3	965	58,4	3,5	2,0	5,0	<0,001	Crescente
Goiás	173	31,6	1.574	51,5	2.122	66,4	5,6	4,0	7,1	<0,001	Crescente
Distrito Federal	14	7,4	583	18,7	580	36,1	12,6	9,7	15,6	<0,001	Crescente
**Brasil**	6.539	28,4	48.019	45,1	48.800	54,6	5,1	3,1	7,0	<0,001	Crescente

Notas: (i) números absolutos e relativos de vínculos de trabalhos precários apresentados para os anos de 2010, 2016 e 2023; (ii) análise de tendência temporal realizada para o período 2010-2023; *n = Número absoluto; ^†^% = Percentual; ^‡^VPA = Variação percentual anual; ^§^IC95% = Intervalo de confiança de 95%; ^||^LI =Limite inferior; ^¶^LS = Limite superior; **Valor de *p* = Valor de probabilidade

A tendência do percentual de vínculos de trabalho precário na ATS foi crescente no Brasil (VPA= 6,0%; IC95%=5,1-6,8%) e em todas as regiões do país, sendo que a região Norte (VPA=10,2%; IC95%=7,4-13,1%) apresentou o maior percentual de incremento. Já entre as unidades da federação, 22 (81,5%) apresentaram tendência crescente, enquanto cinco (18,5%) apresentaram tendência estacionária (Tabela 3).

**Tabela 3-:** Tendência do percentual (%) de vínculos de trabalho precário de enfermeiros na Atenção Terciária à Saúde, com base no tipo de vínculo, segundo unidades da federação e regiões do Brasil, 2010-2023 (n = 23.758.660)

Regiões e Unidades da Federação	Número e percentual de vínculos de trabalho precário							Resultados regressão de Prais-Winsten			
	**2010**		**2016**		**2023**		**VPA** ^‡^	**IC95%** ^§^		Valor de *p* **	**Tendência**
	n*	**%** ^†^	n*	**%** ^†^	n*	**%** ^†^		**LI** ^||^	**LS** ^¶^		
**Região Norte**	503	14,7	2.562	37,7	8.610	54,0	1,2	7,4	13,1	<0,001	Crescente
Rondônia	22	7,9	140	17,3	888	39,6	11,9	7,4	16,7	<0,001	Crescente
Acre	8	2,3	56	10,4	230	28,7	17,4	8,9	26,4	0,001	Crescente
Amazonas	82	12,1	705	56,5	2.180	63,8	1,9	5,8	16,2	<0,001	Crescente
Roraima	46	28,9	33	8,7	111	10,9	-8,3	-16,0	0,1	0,055	Estacionária
Pará	200	14,7	1.077	48,3	3.249	63,7	11,8	6,2	17,7	0,001	Crescente
Amapá	15	11,1	14	4,1	674	58,9	14,7	-5,9	39,8	0,159	Estacionária
Tocantins	130	27,5	537	42,9	1.278	58,1	8,1	5,2	11,2	<0,001	Crescente
**Região Nordeste**	3.648	24,1	11.564	42,9	34.643	56,6	6,5	4,7	8,4	<0,001	Crescente
Maranhão	778	58,5	2.085	72,5	5.105	71,2	1,3	-0,2	2,8	0,086	Estacionária
Piauí	144	23,0	583	49,3	1.872	61,9	8,7	5,3	12,2	<0,001	Crescente
Ceará	327	13,5	1.089	36,7	4.839	52,4	1,8	6,9	14,9	<0,001	Crescente
**Rio Grande do Norte**	129	13,1	193	12,5	1.110	32,5	7,4	1,0	14,2	0,028	Crescente
Paraíba	501	36,2	1.340	53,5	2.901	63,7	4,2	2,6	5,9	<0,001	Crescente
Pernambuco	440	14,1	2.509	41,2	5.684	48,8	9,2	4,6	14,1	0,001	Crescente
Alagoas	49	12,6	309	30,1	1.387	50,5	1,4	8,1	12,9	<0,001	Crescente
Sergipe	102	20,9	243	23,4	998	39,6	6,9	2,4	11,6	0,006	Crescente
Bahia	1.178	26,9	3.213	41,4	10.747	63,8	6,6	5,3	7,8	<0,001	Crescente
**Região Sudeste**	7.119	19,1	19.190	29,0	37.935	34,1	4,3	3,3	5,3	<0,001	Crescente
Minas Gerais	1.198	22,0	2.151	19,5	4.354	20,8	0,8	-1,2	2,9	0,408	Estacionária
Espírito Santo	148	16,2	630	29,0	1.704	38,2	6,6	3,8	9,5	<0,001	Crescente
Rio de Janeiro	2.165	22,8	6.455	39,6	14.286	51,7	6,4	4,6	8,1	<0,001	Crescente
São Paulo	3.608	16,8	9.954	27,1	17.591	30,2	3,9	2,7	5,2	<0,001	Crescente
**Região Sul**	734	8,0	2.294	12,3	5.948	18,7	8,1	6,9	9,3	<0,001	Crescente
Paraná	428	13,6	1.112	16,4	2.487	21,5	5,2	2,7	7,7	0,001	Crescente
Santa Catarina	126	8,4	486	13,4	2.052	28,8	1,9	9,6	12,2	<0,001	Crescente
Rio Grande do Sul	180	4,0	696	8,4	1.409	10,8	8,6	5,0	12,4	<0,001	Crescente
**Região Centro-Oeste**	916	20,7	2.634	31,7	9.941	50,7	8,2	6,8	9,7	<0,001	Crescente
Mato Grosso do Sul	173	32,4	295	21,8	733	23,3	-2,5	-6,1	1,3	0,176	Estacionária
Mato Grosso	227	32,4	549	35,2	2.427	64,5	5,5	3,4	7,7	<0,001	Crescente
Goiás	285	24,6	1.305	56,6	4.477	73,4	8,9	5,7	12,2	<0,001	Crescente
Distrito Federal	231	11,3	485	15,7	2.304	34,9	13,2	6,8	19,9	0,001	Crescente
**Brasil**	12.920	18,6	38.244	30,1	97.077	40,5	6,0	5,1	6,8	<0,001	Crescente

Notas: (i) números absolutos e relativos de vínculos de trabalhos precários apresentados para os anos de 2010, 2016 e 2023; (ii) análise de tendência temporal realizada para o período 2010-2023; *n = Número absoluto; ^†^% = Percentual; ^‡^VPA = Variação percentual anual; ^§^IC95% = Intervalo de confiança de 95%; ^||^LI =Limite inferior; ^¶^LS = Limite superior; **Valor de *p* = Valor de probabilidade

A Figura 2 mostra a distribuição espacial do percentual PVT de enfermeiros nos municípios brasileiros em 2010, 2016 e 2023, segundo o nível de atenção à saúde. É possível observar um aumento do número de municípios com alto percentual de vínculos de trabalho precário no período analisado. Entretanto, este aumento é mais visível para a ATS. Verifica-se também que as regiões Norte e Nordeste, em 2023, apresentaram os maiores percentuais de vínculos de trabalho precário de enfermeiros na APS e ATS.

**Figura 2-:** Distribuição espacial do percentual (%) de vínculos de trabalhos precários de enfermeiros, com base no tipo de vínculo, segundo nível de atenção à saúde nos municípios do Brasil, 2010, 2016 e 2023

Notas: (i) municípios da cor branca indicam dados faltantes para os vínculos profissionais no CNES e (ii) número de observações (n) incluídas na análise de distribuição espacial, sendo: (ii.1): Atenção Primária à Saúde: 105.587 (anos de 2010, 2016 e 2023); (ii.2): Atenção Secundária à Saúde: 103.358 (anos de 2010, 2016 e 2023); (ii.3): Atenção Terciária à Saúde: 148.241 (anos de 2010, 2016 e 2023)

Os resultados mostraram o aumento da precarização do trabalho dos enfermeiros no Brasil, com base no tipo de vínculo e a necessidade de estratégias de desprecarização no SUS.

## Discussão

Os resultados deste estudo indicaram uma tendência crescente da PVT de enfermeiros em todos os níveis de atenção à saúde no Brasil, com os maiores incrementos nas ASS e ATS. Para a APS, a tendência de aumento da PVT ocorreu em todas as regiões, exceto no Sul. Nas ASS e a ATS, houve um crescimento da PVT em todas as regiões do país. Independentemente do nível de atenção, a maioria das unidades da federação apresentou uma tendência crescente. Em 2023, a APS e ASS apresentaram os maiores percentuais de vínculos de TP quando comparado à ATS. Além disso, a análise espacial mostrou um aumento no número de municípios com elevado percentual de vínculos de TP de enfermeiros, especialmente na ATS.

A maioria das investigações que analisou a PVT esteve focada em analisar o tipo de vínculo como *proxy* do indicador de TP^(6,30-32)^, abordagem similar à deste estudo. Em geral, os estudos associaram alguns vínculos ao TP, uma vez que apresentavam baixo nível de rendimento, redução do número de direitos e proteção jurídica do emprego, sendo algumas delas: os profissionais autônomos diretos ou intermediados por organização da sociedade civil, entidade filantrópica ou sem fins lucrativos, cooperativa, empresa privada, empresa não governamental e organização social, além de bolsistas, profissionais em cargos comissionados, consultores e aqueles com contrato por prazo determinado^(30-32)^. Entretanto, outras abordagens consideraram aspectos multidimensionais para mensurar a precariedade dos vínculos trabalhistas, incluindo o uso de escalas amplas^(32-35)^.

Ademais, não foram encontradas pesquisas de tendência e com a mesma abordagem metodológica para a comparação com esta investigação. No entanto, os resultados foram similares a um estudo conduzido com dados de 2007 a 2021, que mostrou o aumento do percentual de vínculos de TP em profissionais de educação física com atuação no SUS, o qual identificou que a APS apresentou o maior número de cadastros de profissionais com vínculos de trabalho precário, inverso ao que ocorre na ASS e ATS^(6)^. Ainda, houve outra investigação que analisou dados do Programa de Melhoria do Acesso da Qualidade da Atenção Básica e que mostrou uma alta proporção de enfermeiro com vínculos de TP, tais como com contratos temporários ou intermediados por Organizações Sociais, além de uma tendência de retração de vínculos estáveis na APS^(11)^. Os resultados desse estudo e as evidências anteriores sugerem que a PVT na APS é um problema emergente e crescente no Brasil.

Destaca-se que o cenário crescente da PVT de enfermeiros na APS no país é uma preocupação, pois a APS atua como eixo norteador do cuidado e modelo prioritário para organização do SUS. Além do mais, esses espaços caracterizam-se por ações individuais e coletivas que incluem a promoção e a proteção da saúde, a prevenção de doenças, o diagnóstico, o tratamento, a reabilitação e a manutenção da saúde^(36)^. O impacto direto da PVT na APS é a alta rotatividade dos enfermeiros, o que gera sobrecarga de trabalho para os demais profissionais, comprometimento no estabelecimento de vínculo com a população atendida e redução da qualidade assistencial^(11,30)^.

Em suma, o presente estudo mostrou maior percentual de incremento dos vínculos de TP na ASS e ATS quando comparado à APS. Não foram encontrados estudos de tendência que compararam as variações percentuais segundo o nível de atenção. No entanto, o estudo que analisou a PVT em profissionais da educação física mostrou o aumento do número de cadastros de profissionais com vínculos precários na ASS e ATS quando comparado à APS^(6)^. Algumas hipóteses para esse incremento mais acentuado na ASS e ATS podem incluir a participação público-privada mais tardia nesses níveis de atenção, abrangendo o aumento dos contratos com Organizações Sociais na atenção especializada^(6)^. Além disso, a partir de 2020, a pandemia da doença causada pelo novo coronavírus (COVID-19) exigiu novas contratações pelos gestores, frente à sobrecarga dos serviços de saúde, sobretudo na atenção especializada^(2)^. Esse aspecto pode ter contribuído para o maior incremento da PVT de enfermeiros na atenção especializada quando comparado à APS^(15)^.

Ainda, verificou-se o maior percentual de vínculos de TP de enfermeiros nas regiões Norte e Nordeste. Esse resultado também foi observado em profissionais da educação física^(6)^. Outros estudos mostram que as regiões Norte e Nordeste concentram os menores municípios do país e as condições de infraestrutura de saúde menores quando comparadas às demais regiões^(22-23)^. Por isso, esses fatores, entre outros, geram maior dificuldade de atrair e reter profissionais de saúde qualificados^(37-38)^ e contribuir para a maior busca de formas de contratação precárias nessas regiões^(6)^. Alguns indicadores podem apontar possíveis impactos da PVT na fixação de profissionais e atenção à saúde nessas localidades; por exemplo, as regiões Norte e Nordeste apresentam a menor razão de profissionais de saúde por habitante e menores percentuais de acesso aos serviços de saúde em comparação às demais regiões^(39-40)^.

O alto percentual de vínculos de TP em todos os níveis de atenção à saúde e sua tendência crescente ainda persiste como um problema para o sistema de saúde, mesmo com as políticas e iniciativas para seu combate implementadas pelo Ministério da Saúde, como o DesprecarizaSUS^(19)^. Por conseguinte, a tendência de aumento do percentual de vínculos de trabalho precário pode estar relacionada a um conjunto de fatores, tais como os problemas de subfinanciamento do SUS^(12)^.

Do ponto de vista operacional, as práticas de precarização visam permitir a flexibilidade e reduzir custos para os serviços de saúde^(41)^. No entanto, alguns estudos mostraram que o alto percentual de PVT afeta a organização da gestão dos serviços de saúde, reduz a ampliação da cobertura das ações e serviços e também a qualidade e integralidade da assistência, podendo impactar em desfechos negativos aos usuários e redução do acesso aos serviços^(2,42)^. Além disso, a mercantilização e descartabilidade da FTS, bem como a alteração dos padrões de gestão e organização do trabalho ocasionada pela PVT, igualmente levam à vulnerabilidade, aumento de riscos relacionados às condições de segurança e saúde no trabalho, expondo os profissionais aos diversos agravos de saúde físicos e mentais. Ademais, o isolamento resultante dessa prática, além da desvalorização advinda da mesma, pode afetar negativamente a solidariedade de classe, levando ao arrefecimento da força sindical^(43)^.

Além do mais, este estudo apresentou limitações, como a qualidade dos dados disponíveis no CNES, que pode levar à subestimação ou superestimação do indicador de percentual de vínculos de trabalho precário. Por outro lado, a base de dados possui confiabilidade^(44)^ e é frequentemente utilizada em estudos da área. O estudo focou na investigação da PVT, não abrangendo outras dimensões da precarização do trabalho na saúde, como multiplicidade de vínculos, baixa remuneração, falta de infraestrutura de trabalho e descanso, violência, assédio, esforço repetitivo e as consequências da PVT para a saúde dos trabalhadores e a qualidade da assistência aos usuários^(2,41)^. A base de dados também não permitiu avaliar o número de vínculos de um mesmo enfermeiro, devido à ausência de identificação dos participantes, logo, não foi possível analisar a tendência temporal de vínculos duplos ou triplos desses profissionais. Por fim, a presente investigação incluiu dados antes e durante a pandemia de COVID-19, período em que muitos enfermeiros foram contratados para atender às demandas emergenciais de saúde, em sua maioria por meio de contratos temporários e, por vezes, sem garantias de direitos sociais. Esse fato pode ter contribuído para reduzir a precisão na apuração da tendência temporal.

No entanto, este estudo apresentou avanços importantes no conhecimento da PVT de enfermeiros, explorando tendências temporais dessa dimensão desagregada por regiões, unidades da federação e nível de atenção à saúde, subsidiando o monitoramento e a necessidade de políticas públicas que promovam a desprecarização do trabalho no SUS.

Novas investigações podem analisar os determinantes da PVT de enfermeiros, como a análise de variáveis de natureza socioeconômica [por exemplo, Produto Interno Bruto (PIB) *per capita*, média salarial, gasto com saúde *per capita*, etc.], infraestrutura (como os tipos de unidades), entre outras, contribuindo para compreender o fenômeno da PVT, especialmente em determinadas regiões e estados do Brasil. Para além desses determinantes, é importante compreender como a precarização afeta alguns desfechos, seja de eficiência dos sistemas e serviços de saúde ou da qualidade assistencial, pois compreender a precarização da FTS exige uma abordagem multidimensional. Além dos tipos de vínculos analisados neste estudo, pesquisas futuras podem incorporar outras fontes secundárias para examinar componentes adicionais, como rendimentos médios, multiplicidade de vínculos e adoecimento dos trabalhadores, possibilitando a construção de um índice multidimensional da precarização. Existe uma amplitude de evidências que mostram efeitos adversos da precarização, na saúde do trabalhador da saúde, no nível do indivíduo, como adoecimento e vulnerabilidade socioeconômica. Todavia, é importante investigar, ao nível ecológico, como a precarização pode influenciar elementos como a retenção, a densidade de profissionais e a qualidade da assistência em saúde.

## Conclusão

Este estudo mostrou tendência crescente do PVT de enfermeiros no Brasil, independentemente do nível de atenção e da região geográfica, com base no tipo de vínculo. Além disso, as regiões Norte e Nordeste apresentaram os maiores percentuais de vínculos de TP, sugerindo desigualdades na PVT no país. Os resultados mostraram aumento da precarização de vínculos de trabalho de enfermeiros e a necessidade de estratégias de desprecarização no SUS.

## Data Availability

O conjunto de dados deste artigo está disponível em https://drive.google.com/drive/folders/1rEtK9rJJKjRWVFd6OByr_MPx8m9cxkHe

# Translation: Tendencias y distribución espacial de la precarización del trabajo de los enfermeros en Brasil según el tipo de vínculo laboral*

**DOI:** 10.1590/1518-8345.7680.4643

**Authors:** Érika Carvalho de Aquino, Rafael Alves Guimarães, Daniel do Prado Pagotto, Joseane Aparecida Duarte, Antônio Isidro da Silva, Cândido Vieira Borges

**Affiliations:** 1 Universidade Federal de Goiás, Instituto de Patologia Tropical e Saúde Pública, Goiânia, GO, Brasil.; 2 Universidade Federal de Goiás, Faculdade de Enfermagem, Goiânia, GO, Brasil.; 3 Universidade de Brasília, Brasília, DF, Brasil.; 4 Universidade Federal da Bahia, Salvador, BA, Brasil.; 5 Universidade Federal de Goiás, Faculdade de Administração, Ciências Contábeis e Ciências Econômicas, Goiânia, GO, Brasil.

**Keywords:** Fuerza de Trabajo, Condiciones de Trabajo, Trabajo Precario, Seguridad del Empleo, Enfermeras y Enfermeros, Niveles de Atención de Salud

## Abstract

analizar la tendencia y la distribución espacial de la precarización del trabajo de los enfermeros en Brasil, según el tipo de vínculo, entre 2010 y 2023, de acuerdo con el nivel de atención a la salud.

estudio ecológico con datos extraídos del *Cadastro Nacional de Estabelecimentos de Saúde*. El indicador analizado fue el porcentaje de vínculos laborales precarios. La tendencia temporal fue evaluada mediante el modelo de regresión de Prais-Winsten y la distribución espacial, mediante mapas coropléticos.

se observó una tendencia creciente en la precarización del trabajo de los enfermeros en Brasil según el tipo de vínculo, independientemente del nivel de atención a la salud. La región Norte presentó los mayores porcentajes de aumento del indicador en la Atención Primaria de Salud, Atención Secundaria de Salud y Atención Terciaria de Salud. Las regiones Norte y Nordeste presentaron los mayores números de municipios con alto porcentaje de vínculos laborales precarios.

la precarización de los vínculos laborales de los enfermeros, según el tipo de vínculo, mostró una tendencia creciente en todos los niveles de atención a la salud, siendo más elevada en la Atención Primaria de Salud y con mayor incremento en la Atención Terciaria de Salud dentro del horizonte temporal analizado.

## Destacados

**(1)** La precarización de los vínculos laborales de los enfermeros aumentó sustancialmente. **(2)** La precarización de los vínculos fue alta en la Atención Primaria de Salud. **(3)** Las regiones Norte y Nordeste presentaron mayor precarización de los vínculos. **(4)** La Atención Terciaria de Salud registró un mayor incremento en la precarización de los vínculos.

## Introducción

La Precarización de los Vínculos Laborales (PVL) representa un grave problema global y tiene consecuencias adversas para la salud de los trabajadores, la gestión y la calidad de la atención en salud^(1-2)^, disminuyendo la capacidad de los sistemas de salud para alcanzar sus objetivos, especialmente la Cobertura Universal de Salud^(3)^. El Trabajo Precario (TP) es una construcción multidimensional caracterizada por condiciones de empleo de baja calidad, incluyendo la corta duración del vínculo laboral, vínculos temporales, baja remuneración, falta de derechos y protección a los trabajadores e inseguridad laboral^(4-5)^.

En Brasil, la PVL representa un desafío para el *Sistema Único de Saúde* (SUS), con una tendencia creciente^(6)^, potenciada por políticas neoliberales, así como por el aumento de la tercerización y la flexibilización de los contratos^(7)^, como resultado de cambios legislativos y restricciones presupuestarias del sistema de salud, tales como las Enmiendas Constitucionales número 19 de 1998^(8)^ y número 95 de 2016^(9)^ y el establecimiento de asociaciones público-privadas para la constitución de Redes de Atención a la Salud^(7)^. Estas medidas agravaron las desigualdades regionales y perjudicaron la asignación de profesionales calificados en los territorios^(10)^. Otros estudios muestran que la flexibilización de los contratos, con énfasis en la tercerización y subcontratación, resultó en vínculos laborales más precarios, comprometiendo la calidad de los servicios prestados en el SUS^(11-12)^.

Una de las categorías profesionales que actúan en el SUS más impactadas por la PVL es la enfermería, compuesta por enfermeros, técnicos y auxiliares de enfermería^(2-13)^. La enfermería constituye la mayor Fuerza de Trabajo en Salud (FTS) del país^(14)^, desempeñando un papel fundamental en el funcionamiento del SUS en todos los niveles de atención. A pesar de ello, muchos profesionales de enfermería enfrentan condiciones laborales precarias, como vínculos temporales sin garantías de derechos sociales, baja remuneración y sobrecarga^(15-16)^. Esta realidad compromete la capacidad del sistema para ofrecer servicios de salud de calidad y retener profesionales en regiones de baja densidad poblacional^(16)^.

No obstante, no se ha realizado ningún estudio nacional amplio con datos poblacionales que evalúe las tendencias de la PVL de enfermeros, especialmente desagregado por regiones y unidades federativas. Además, este fenómeno puede comportarse de manera distinta entre los niveles de atención a la salud, siendo la Atención Primaria de Salud (APS) la que abarca la mayor parte de los vínculos precarios^(6)^. Por tanto, también existe una laguna en la literatura sobre las tendencias de la PVL de enfermeros según el nivel de atención a la salud. Ante este escenario, el estudio de las tendencias y de la distribución espacial de la PVL en enfermeros se muestra fundamental y urgente en el país, ya que está alineado con las agendas internacionales, como el octavo Objetivo de Desarrollo Sostenible de la Organización de las Naciones Unidas^(17)^ y la Estrategia Mundial de Recursos Humanos en Salud de la Organización Mundial de la Salud^(18)^, además de contribuir al diseño de estrategias para la desprecarización de los vínculos laborales en salud, como el Programa Nacional de Desprecarización del Trabajo en el SUS^(19)^.

De este modo, el objetivo de este estudio fue analizar la tendencia y la distribución espacial de la precarización del trabajo de los enfermeros en Brasil, según el tipo de vínculo, entre 2010 y 2023, de acuerdo con el nivel de atención a la salud.

## Método

### Diseño del estudio

Estudio ecológico de series temporales y de base poblacional. El presente manuscrito fue elaborado utilizando adaptaciones del *Revised Standards for Quality Improvement Reporting Excellence* (SQUIRE 2.0)^(20)^.

### Contexto

El estudio fue realizado en Brasil, con base en los datos de todos los municipios brasileños entre 2010 y 2023. Este período fue seleccionado por permitir el análisis de una serie histórica más amplia, proporcionando insumos para la discusión de diversas legislaciones, programas y políticas públicas que contribuyeron a la PVL en dicho intervalo. Además, este recorte temporal incluyó tanto el período anterior como el de la pandemia de COVID-19, lo que posibilitó la evaluación de cambios en el contexto de los vínculos laborales durante y después de la crisis sanitaria, en la que muchos enfermeros fueron sometidos a vínculos de trabajo precarios^(12)^.

Cabe destacar que el país presentaba una población estimada de 203.080.756 millones de habitantes en 2022, según el último censo demográfico del *Instituto Brasileiro de Geografia e Estatística*, distribuidos en 5.568 municipios^(21)^, agrupados en 26 unidades federativas y el Distrito Federal. Las regiones presentan características demográficas, sociales y económicas distintas, además de diferencias en el contingente de enfermeros y en el número de dispositivos e inversiones en las Redes de Atención a la Salud^(22-23)^.

### Participantes

Fueron incluidos en el estudio todos los vínculos laborales de enfermeros que trabajaban en servicios de salud en Brasil registrados en el *Cadastro Nacional de Estabelecimentos de Saúde* (CNES) entre 2010 y 2023, independientemente del nivel de actuación (primario, secundario y terciario).

### Fuente de datos y variables

Los datos sobre los vínculos laborales de enfermeros que actuaban en los servicios de salud en Brasil fueron extraídos de los microdatos del CNES, según el diseño de los archivos de profesionales, disponibles públicamente. La extracción se realizó mediante el protocolo de transferencia de archivos del Departamento de Informática del SUS, el 10 de enero de 2024.

El CNES es un Sistema de Información en Salud que contiene datos sobre la estructura física de las unidades de salud, los servicios de salud disponibles y los profesionales vinculados a los establecimientos de salud del territorio nacional, en todos los niveles de gestión (nacional, estatal y municipal), estén o no vinculados al SUS. Los datos se ingresan por medio del llenado de formularios con variables sobre la estructura física, los equipos, la FTS, entre otros elementos^(24)^.

Se extrajeron del CNES los siguientes datos: el código del municipio del establecimiento de salud, código CNES de la unidad, tipo de unidad, el código brasileño de ocupaciones (CBO), tipo de vínculo del profesional y subtipo de vínculo del profesional. Se filtraron los CBO cuya familia comenzaba con el código 2235, correspondiente a los enfermeros y afines.

Además, cabe destacar que los microdatos representan el nivel más desagregado del CNES, lo que significa que cada observación corresponde a un vínculo laboral. Estos fueron organizados de acuerdo con el tipo y subtipo de vínculo, extraídos del campo “vínculo laboral con el establecimiento”. Según un estudio anterior^(6)^, los vínculos fueron clasificados como: (i) protegidos: servidor público, servidor público cedido a la iniciativa privada, celetista (empleado regido por la legislación laboral brasileña, la CLT - Consolidación de las Leyes del Trabajo), empleado público y estatutario; (ii) precarios: autónomos (incluidos aquellos con vínculo directo, sin intermediación, o aquellos con vínculo indirecto, cuando son intermediados por instituciones o entidades como cooperativas, Organizaciones de la Sociedad Civil de Interés Público, entidades filantrópicas y/o sin fines de lucro, empresas privadas, Organizaciones No Gubernamentales y Organizaciones Sociales), becarios, cooperativistas, contratos verbales o informales, voluntarios, cargos de confianza, contratos temporales o por tiempo determinado; (iii) otros: propietarios, pasantes y residentes y (iv) sin información: tipo de vínculo ausente en el CNES.

De este modo, los vínculos laborales fueron clasificados de acuerdo con el nivel de atención en: (i) APS^(6,25)^, que incluyó los siguientes códigos del CNES: 01 – puesto de salud; 02 – centro de salud/unidad básica; 32 – unidad móvil fluvial; 40 – unidad móvil terrestre; 71 – centro de apoyo a la salud de la familia; 72 – unidad de atención a la salud indígena y 74 – polo academia de la salud; (ii) Atención Secundaria de Salud (ASS), que comprendió los siguientes códigos: 04 – policlínica; 15 – unidad mixta; 20 – sala de urgencias general; 21 – sala de urgencias especializada; 22 – consultorio aislado; 36 – clínica/centro especializado; 39 – unidad de apoyo diagnóstico y terapéutico; 42 – unidad móvil de nivel prehospitalario en el área de urgencias; 61 – centro de parto normal – aislado; 62 – hospital de día – aislado; 69 – centro de atención hemoterapéutica y/o hematológica; 70 – CAPS; 73 – atención de urgencias; 83 – polos de prevención de enfermedades y daños y promoción de la salud y (iii) Atención Terciaria de Salud (ATS), que incluyó los códigos 05 – hospital general y 07 – hospital especializado. De este modo, se excluyeron los vínculos asociados a los demás establecimientos de salud.

Estudios anteriores también utilizaron análisis sobre los tipos de vínculos laborales para investigar la precarización^(6,11,25)^. Sin embargo, la clasificación adoptada en este estudio fue seleccionada por ser categórica en relación con los tipos de vínculo y sus respectivos grados de precarización^(6)^, además de ser compatible con la estructura de datos utilizada, el CNES.

El indicador analizado fue el porcentaje (%) de vínculos laborales precarios de enfermeros. Este indicador fue calculado, para cada año, mediante la siguiente fórmula:

$$\frac{Númerodevínculoslaboralesprecariosendeterminadalocalidadyperíodo}{Númerototaldevínculosenlamismalocalidadyperíodo}x100$$

El horizonte temporal fue de 2010 a 2023. Se analizaron los números de vínculos correspondientes al mes de junio de cada año, por tratarse de la mitad del año, además de presentar menor influencia de cambios en la gestión durante ese período y ser congruente con indicadores poblacionales anuales que también hacen referencia a dicho mes.

### Análisis estadístico

Inicialmente, se realizaron análisis descriptivos mediante el número absoluto de la PVL y del porcentaje (%) de vínculos laborales precarios.

A continuación, se analizaron las tendencias temporales mediante el modelo de regresión lineal de Prais-Winsten. Antes de la inclusión en los modelos de regresión, se realizó la transformación logarítmica en base 10 de las tasas para reducir la heterogeneidad de la varianza de los residuos y contribuir a la evaluación de la tendencia temporal^(26)^. La variable dependiente (Y) utilizada fue el porcentaje (%) de vínculos laborales precarios, mientras que la variable independiente (X) fue el año de la serie temporal. La ecuación de regresión se definió como ^(26)^, siendo los porcentajes (%) de vínculos laborales precarios de enfermeros tras la transformación logarítmica, el intercepto o constante de la regresión, el coeficiente de pendiente de la recta y el error aleatorio. La variable *t* estima los tiempos del conjunto de datos {*t*_1_, ..., *t*_14_}, en este caso *t*_1_ = 2010 y *t*_14_ = 2023.

Mediante los modelos de regresión, fue posible obtener el valor del coeficiente de pendiente de la recta () y los errores estándar (EE). Con estos parámetros, se calculó la Variación Porcentual Anual (VPA), según la siguiente fórmula^(26)^:

$$VPA=\left(1+10^{\beta_{1}}\right)*100$$

siendo el coeficiente de pendiente de la recta.

Los límites inferior y superior del Intervalo de Confianza del 95% (IC95%) de la VPA fueron calculados mediante la fórmula^(26)^:

$$IC95\%=\left(1+10^{\beta_{1}\left(t*EP\right)}\right)*100$$

siendo el coeficiente de pendiente de la recta obtenido en el modelo de regresión, *t* el valor que presenta la distribución *t* de Student con 13 grados de libertad (*n*-1) a un IC95% bilateral y EP el error estándar de la estimación de obtenida en la regresión.

Los análisis se realizaron de forma desagregada según el nivel de atención: (i) APS, (ii) ASS y (iii) ATS para Brasil, las cinco grandes regiones y las 27 unidades federativas, constituyendo un total de 99 series temporales analizadas. Además, se realizaron análisis de la distribución espacial del indicador según el nivel de atención para los años 2010, 2016 y 2023. Se construyeron mapas coropléticos utilizando como unidad de análisis el número de municipios brasileños (n=5.568).

Las extracciones y el procesamiento de los datos se realizaron utilizando el lenguaje *Structured Query Language* (SQL). Los análisis descriptivos y los modelos de series temporales se realizaron utilizando el lenguaje R, con la interfaz RStudio^(27)^. Los análisis de distribución espacial se llevaron a cabo con el *software* QGIS (*QGIS Development Team*, 2024)^(28)^.

### Aspectos éticos

No fue necesaria la presentación al Comité de Ética en Investigación, ya que se utilizaron exclusivamente microdatos secundarios y de acceso público, por medio de la herramienta de transferencia de archivos del DATASUS, conforme a la exención prevista en la Resolución nº 510/2016 del Consejo Nacional de Salud^(29)^.

## Resultados

Entre 2010 y 2023, se registraron 47.411.575 vínculos laborales de enfermeros en el CNES. De estos, se excluyeron 2.629.306 (5,54%) registros relacionados con unidades de salud cuyos tipos no formaron parte del análisis. De esta forma, se analizaron 44.782.269 vínculos laborales, lo que representa el 94,46% del total de vínculos en el período. De estos, 12.186.668 (27,21%) correspondían a unidades de la APS, 8.836.941 (19,73%) a la ASS y 23.758.660 (53,05%) a la ATS.

La Figura 1 presenta la evolución del porcentaje (%) de vínculos laborales precarios de enfermeros, según el nivel de atención y regiones. En Brasil, el porcentaje de vínculos precarios fue similar en la APS y ASS y menor en la ATS. Este patrón se repite en las regiones Sudeste y Nordeste. Sin embargo, la región Sur presenta el mayor porcentaje de vínculos precarios en la ASS en comparación con los demás niveles de atención, mientras que la región Norte presenta el mayor porcentaje en la APS. En la región Centro-Oeste, los porcentajes de vínculos precarios entre los niveles de atención se aproximan a lo largo del tiempo, con valores similares a partir de 2021.

Como resultado, Brasil presentó una tendencia creciente en el porcentaje de PVL en la APS (VPA = 2,8%; IC95% = 2,1–3,4%). Todas las regiones presentaron una tendencia creciente del indicador en la APS, excepto la región Sur. La región Norte fue la que mostró el mayor porcentaje de incremento (VPA = 4,8%; IC95% = 3,1–6,6%). Entre las unidades federativas, 21 (77,8%) presentaron una tendencia creciente, mientras que seis (22,2%) mostraron una tendencia estacionaria en el porcentaje de PVL de enfermeros en la APS (Tabla 1).

**Figura 1-:** Porcentaje (%) de vínculos laborales precarizados de enfermeros, según nivel de atención a la salud y regiones de Brasil, 2010–2023 (n = 44.782.269)

Nota: Número de observaciones (n) incluidas en el análisis de las series históricas: (ii.1): Atención Primaria de Salud: 12.186.668; (ii.2): Atención Secundaria de Salud: 8.836.941 y (ii.3): Atención Terciaria de Salud: 23.758.660.

**Tabla 1-:** Tendencia del porcentaje (%) de vínculos laborales precarios de enfermeros en la Atención Primaria de Salud, según el tipo de vínculo, por unidades federativas y regiones de Brasil, 2010-2023 (n = 12.186.668)

**Regiones y Unidades de la Federación**	**Número y porcentaje de vínculos laborales precarios**							Regresión de Prais-Winsten			
	**2010**		**2016**		**2023**		**VPA** ^‡^	**IC95%** ^§^		Valor de *p* **	**Tendencia**
	n*	**%** ^†^	n*	**%** ^†^	n*	**%** ^†^		**LI** ^||^	**LS** ^¶^		
**Región** **Norte**	1.758	39,1	3.717	59,3	6.468	72,4	4,8	3,1	6,6	<0,001	Creciente
Rondônia	65	14,2	108	17,9	264	33,5	7,6	4,8	10,5	<0,001	Creciente
Acre	58	20,8	124	32,7	211	43,7	6,1	3,6	8,7	<0,001	Creciente
Amazonas	506	53,4	976	72,1	1.730	85,9	3,7	2,8	4,6	<0,001	Creciente
Roraima	96	61,1	152	56,3	267	60,7	0,1	-3,1	3,4	0,947	Estacionaria
Pará	733	39,6	1.746	67,8	2.744	78,2	5,3	2,7	8,0	0,001	Creciente
Amapá	9	3,6	182	56,0	419	74,3	25,5	7,0	47,1	0,010	Creciente
Tocantins	291	52,7	429	56,0	833	73,9	3,6	2,7	4,5	<0,001	Creciente
**Región** **Nordeste**	7.865	45,4	11.689	57,5	18.128	67,6	3,1	2,4	3,8	<0,001	Creciente
Maranhão	1.376	60,9	1.991	73,4	2.850	82,9	2,3	1,6	3,0	<0,001	Creciente
Piauí	258	21,4	555	36,2	983	49,1	7,0	5,3	8,7	<0,001	Creciente
Ceará	739	27,3	1.684	51,5	2.793	62,4	6,5	4,1	8,9	<0,001	Creciente
Rio Grande do Norte	685	62,4	754	57,9	1.033	63,1	0,5	-0,1	1,0	0,101	Estacionaria
Paraíba	739	46,6	883	49,9	1.347	59,3	2,3	1,5	3,1	<0,001	Creciente
Pernambuco	1.416	60,0	1.997	69,5	2.560	70,7	1,2	0,3	2,0	0,013	Creciente
Alagoas	240	24,5	391	34,5	997	60,7	7,1	5,4	8,7	<0,001	Creciente
Sergipe	130	18,4	247	31,9	775	63,3	11,1	10,2	12,1	<0,001	Creciente
Bahia	2.282	51,8	3.187	64,1	4.790	73,6	2,6	1,6	3,5	<0,001	Creciente
**Región Sudeste**	7.675	38,7	12.451	47,1	19.350	52,1	2,1	1,4	2,8	<0,001	Creciente
Minas Gerais	2.857	44,9	4.408	53,2	6.383	55,0	1,5	0,6	2,3	0,004	Creciente
Espírito Santo	496	41,8	565	47,7	1.207	65,5	3,3	1,6	5,0	0,001	Creciente
Rio de Janeiro	1.540	50,3	2.870	62,6	3.880	67,2	1,9	0,8	3,0	0,003	Creciente
São Paulo	2.782	30,2	4.608	37,2	7.880	44,0	2,9	2,4	3,4	<0,001	Creciente
**Región** **Sur**	1.722	20,3	2.061	18,9	5.531	30,4	3,7	-0,2	7,7	0,065	Estacionaria
Paraná	556	17,7	477	11,6	1.280	19,8	1,0	-4,6	6,9	0,707	Estacionaria
Santa Catarina	530	24,5	600	21,5	1.584	32,7	3,2	-1,4	8,0	0,159	Estacionaria
Rio Grande do Sul	636	20,1	984	24,5	2.667	38,7	5,6	3,3	7,9	<0,001	Creciente
**Región** **Centro-Oeste**	1.369	33,9	2.113	41,7	3.690	47,4	2,6	2,1	3,0	<0,001	Creciente
Mato Grosso do Sul	166	22,4	409	40,9	476	35,2	3,0	0,8	5,2	0,011	Creciente
Mato Grosso	445	50,7	523	43,7	898	50,0	0,4	-1,3	2,2	0,630	Estacionaria
Goiás	734	40,0	1.164	57,0	2.105	71,1	4,2	2,5	5,9	<0,001	Creciente
Distrito Federal	24	4,1	17	2,1	211	12,6	9,2	-7,7	29,3	0,280	Estacionaria
**Brasil**	20.389	37,7	32.031	46,4	53.167	53,8	2,8	2,1	3,4	<0,001	Creciente

Notas: (i) se presentan los números absolutos y relativos de vínculos laborales precarios para los años 2010, 2016 y 2023; (ii) el análisis de tendencia temporal se realizó para el período 2010–2023; *n = Número absoluto; ^†^% = Porcentaje; ^‡^VPA = Variación porcentual anual; ^§^IC95% = Intervalo de confianza del 95%; ^||^LI = Límite inferior; ^¶^LS = Límite superior; **Valor de *p* = Valor de probabilidad

La tendencia en la ASS fue creciente en Brasil (VPA = 5,1%; IC95% = 3,1–7,0%) y en todas las regiones del país. Las regiones Norte (VPA = 5,8%; IC95% = 4,1–7,6%) y Sur (VPA = 5,9%; IC95% = 4,4–7,3%) fueron las que presentaron los mayores aumentos. Entre las unidades federativas, 25 (92,6%) presentaron una tendencia creciente, una (3,7%) presentó una tendencia decreciente y una (3,7%) presentó una tendencia estacionaria (Tabla 2).

**Tabla 2-:** Tendencia del porcentaje (%) de vínculos laborales precarios de enfermeros en la Atención Secundaria de Salud, según el tipo de vínculo, por unidades federativas y regiones de Brasil, 2010–2023 (n = 8.836.941)

**Regiones y Unidades de la Federación**	**Número y porcentaje de vínculo laboral precario**							**Regresión de Prais-Winsten**			
	**2010**		**2016**		**2023**		**VPA** ^‡^	**IC95%** ^§^		Valor de *p* **	**Tendencia**
	n*	**%** ^†^	n*	**%** ^†^	n*	**%** ^†^		**LI** ^||^	**LS** ^¶^		
**Región** **Norte**	474	26,4	2.883	43,8	2.982	56,4	5,8	4,1	7,6	<0,001	Creciente
Rondônia	31	12,8	349	22,9	309	40,8	7,3	4,9	9,7	<0,001	Creciente
Acre	7	9,6	143	15,4	128	40,1	13,1	6,6	19,9	0,001	Creciente
Amazonas	213	38,3	398	52,8	421	48,3	0,8	-3,0	4,8	0,655	Estacionaria
Roraima	23	39,0	119	25,2	69	25,1	-3,8	-6,3	-1,2	0,009	Decreciente
Pará	152	22,8	1.321	54,4	1.530	70,9	9,1	4,8	13,6	0,001	Creciente
Amapá	2	3,0	190	17,9	202	59,4	22,2	14,5	30,4	<0,001	Creciente
Tocantins	46	35,1	363	46,6	323	57,2	2,9	0,2	5,6	0,037	Creciente
**Región** **Nordeste**	2.171	37,1	12.708	60,3	15.307	67,7	4,6	2,3	7,0	0,001	Creciente
Maranhão	287	46,1	1.090	70,2	1.595	69,3	2,8	0,3	5,4	0,034	Creciente
Piauí	148	39,3	801	47,7	809	58,5	2,7	1,4	4,0	0,001	Creciente
Ceará	214	29,4	2.188	57,2	2.349	72,8	7,3	4,9	9,8	<0,001	Creciente
Rio Grande do Norte	181	40,6	767	48,6	906	56,6	2,5	0,9	4,0	0,004	Creciente
Paraíba	171	39,0	1.526	64,8	1.881	66,0	3,9	0,2	7,6	0,040	Creciente
Pernambuco	353	33,4	1.931	67,1	2.222	69,9	5,2	2,2	8,3	0,003	Creciente
Alagoas	96	23,7	831	52,0	1.171	71,8	7,6	5,5	9,8	<0,001	Creciente
Sergipe	92	32,2	427	56,4	439	54,8	5,4	1,6	9,2	0,009	Creciente
Bahia	629	42,3	3.147	61,4	3.935	69,9	3,7	2,2	5,2	<0,001	Creciente
**Región** **Sudeste**	2.956	26,5	22.136	43,2	21.221	52,3	5,1	3,0	7,4	<0,001	Creciente
Minas Gerais	824	31,3	4.986	44,3	4.067	46,8	3,4	1,2	5,8	0,006	Creciente
Espírito Santo	105	32,4	749	29,8	927	51,9	3,3	0,5	6,3	0,025	Creciente
Rio de Janeiro	734	29,3	4.966	57,5	5.500	64,6	4,3	1,6	7,2	0,005	Creciente
São Paulo	1.293	22,7	11.435	37,4	10.727	49,6	6,1	4,7	7,4	<0,001	Creciente
**Región** **Sur**	593	20,8	6.788	27,3	5.400	40,8	5,9	4,4	7,3	<0,001	Creciente
Paraná	187	19,0	2.527	22,4	1.788	35,5	5,8	2,7	8,9	0,001	Creciente
Santa Catarina	160	24,6	1.496	31,0	1.452	43,7	5,7	3,8	7,5	<0,001	Creciente
Rio Grande do Sul	246	20,2	2.765	29,8	2.160	44,2	6,4	5,7	7,1	<0,001	Creciente
**Región** **Centro-Oeste**	345	25,2	3.504	37,2	3.890	50,3	4,8	4,0	5,6	<0,001	Creciente
Mato Grosso do Sul	24	9,4	691	18,1	223	17,5	6,5	2,1	11,1	0,007	Creciente
Mato Grosso	134	35,6	656	39,3	965	58,4	3,5	2,0	5,0	<0,001	Creciente
Goiás	173	31,6	1.574	51,5	2.122	66,4	5,6	4,0	7,1	<0,001	Creciente
Distrito Federal	14	7,4	583	18,7	580	36,1	12,6	9,7	15,6	<0,001	Creciente
**Brasil**	6.539	28,4	48.019	45,1	48.800	54,6	5,1	3,1	7,0	<0,001	Creciente

Notas: (i) se presentan los números absolutos y relativos de vínculos laborales precarios para los años 2010, 2016 y 2023; (ii) el análisis de tendencia temporal se realizó para el período 2010–2023; *n = Número absoluto; ^†^% = Porcentaje; ^‡^VPA = Variación porcentual anual; ^§^IC95% = Intervalo de confianza del 95%; ^||^LI = Límite inferior; ^¶^LS = Límite superior; **Valor de *p* = Valor de probabilidad

La tendencia del porcentaje de vínculos laborales precarios en la ATS fue creciente en Brasil (VPA = 6,0%; IC95% = 5,1–6,8%) y en todas las regiones del país, siendo que la región Norte (VPA = 10,2%; IC95% = 7,4–13,1%) presentó el mayor porcentaje de incremento. Entre las unidades federativas, 22 (81,5%) presentaron una tendencia creciente, mientras que cinco (18,5%) presentaron una tendencia estacionaria (Tabla 3).

**Tabla 3-:** Tendencia del porcentaje (%) de vínculos laborales precarios de enfermeros en la Atención Terciaria de Salud, según el tipo de vínculo, por unidades federativas y regiones de Brasil, 2010–2023 (n = 23.758.660)

Regiones y Unidades de la Federación	Número y porcentaje de vínculo laboral precario							Resultados regresión de Prais-Winsten			
	**2010**		**2016**		**2023**		**VPA** ^‡^	**IC95%** ^§^		Valor de *p* **	**Tendencia**
	n*	**%** ^†^	n*	**%** ^†^	n*	**%** ^†^		**LI** ^||^	**LS** ^¶^		
**Región Norte**	503	14,7	2.562	37,7	8.610	54,0	1,2	7,4	13,1	<0,001	Creciente
Rondônia	22	7,9	140	17,3	888	39,6	11,9	7,4	16,7	<0,001	Creciente
Acre	8	2,3	56	10,4	230	28,7	17,4	8,9	26,4	0,001	Creciente
Amazonas	82	12,1	705	56,5	2.180	63,8	1,9	5,8	16,2	<0,001	Creciente
Roraima	46	28,9	33	8,7	111	10,9	-8,3	-16,0	0,1	0,055	Estacionaria
Pará	200	14,7	1.077	48,3	3.249	63,7	11,8	6,2	17,7	0,001	Creciente
Amapá	15	11,1	14	4,1	674	58,9	14,7	-5,9	39,8	0,159	Estacionaria
Tocantins	130	27,5	537	42,9	1.278	58,1	8,1	5,2	11,2	<0,001	Creciente
**Región Nordeste**	3.648	24,1	11.564	42,9	34.643	56,6	6,5	4,7	8,4	<0,001	Creciente
Maranhão	778	58,5	2.085	72,5	5.105	71,2	1,3	-0,2	2,8	0,086	Estacionaria
Piauí	144	23,0	583	49,3	1.872	61,9	8,7	5,3	12,2	<0,001	Creciente
Ceará	327	13,5	1.089	36,7	4.839	52,4	1,8	6,9	14,9	<0,001	Creciente
Rio Grande do Norte	129	13,1	193	12,5	1.110	32,5	7,4	1,0	14,2	0,028	Creciente
Paraíba	501	36,2	1.340	53,5	2.901	63,7	4,2	2,6	5,9	<0,001	Creciente
Pernambuco	440	14,1	2.509	41,2	5.684	48,8	9,2	4,6	14,1	0,001	Creciente
Alagoas	49	12,6	309	30,1	1.387	50,5	1,4	8,1	12,9	<0,001	Creciente
Sergipe	102	20,9	243	23,4	998	39,6	6,9	2,4	11,6	0,006	Creciente
Bahia	1.178	26,9	3.213	41,4	10.747	63,8	6,6	5,3	7,8	<0,001	Creciente
**Región Sudeste**	7.119	19,1	19.190	29,0	37.935	34,1	4,3	3,3	5,3	<0,001	Creciente
Minas Gerais	1.198	22,0	2.151	19,5	4.354	20,8	0,8	-1,2	2,9	0,408	Estacionaria
Espírito Santo	148	16,2	630	29,0	1.704	38,2	6,6	3,8	9,5	<0,001	Creciente
Rio de Janeiro	2.165	22,8	6.455	39,6	14.286	51,7	6,4	4,6	8,1	<0,001	Creciente
São Paulo	3.608	16,8	9.954	27,1	17.591	30,2	3,9	2,7	5,2	<0,001	Creciente
**Región Sur**	734	8,0	2.294	12,3	5.948	18,7	8,1	6,9	9,3	<0,001	Creciente
Paraná	428	13,6	1.112	16,4	2.487	21,5	5,2	2,7	7,7	0,001	Creciente
Santa Catarina	126	8,4	486	13,4	2.052	28,8	1,9	9,6	12,2	<0,001	Creciente
Rio Grande do Sul	180	4,0	696	8,4	1.409	10,8	8,6	5,0	12,4	<0,001	Creciente
**Región Centro-Oeste**	916	20,7	2.634	31,7	9.941	50,7	8,2	6,8	9,7	<0,001	Creciente
Mato Grosso do Sul	173	32,4	295	21,8	733	23,3	-2,5	-6,1	1,3	0,176	Estacionaria
Mato Grosso	227	32,4	549	35,2	2.427	64,5	5,5	3,4	7,7	<0,001	Creciente
Goiás	285	24,6	1.305	56,6	4.477	73,4	8,9	5,7	12,2	<0,001	Creciente
Distrito Federal	231	11,3	485	15,7	2.304	34,9	13,2	6,8	19,9	0,001	Creciente
**Brasil**	12.920	18,6	38.244	30,1	97.077	40,5	6,0	5,1	6,8	<0,001	Creciente

Notas: (i) se presentan los números absolutos y relativos de vínculos laborales precarios para los años 2010, 2016 y 2023; (ii) el análisis de tendencia temporal se realizó para el período 2010–2023; *n = Número absoluto; ^†^% = Porcentaje; ^‡^VPA = Variación porcentual anual; ^§^IC95% = Intervalo de confianza del 95%; ^||^LI = Límite inferior; ^¶^LS = Límite superior; **Valor de *p* = Valor de probabilidad

La Figura 2 muestra la distribución espacial del porcentaje de PVL de enfermeros en los municipios brasileños en 2010, 2016 y 2023, según el nivel de atención a la salud. Es posible observar un aumento en el número de municipios con un alto porcentaje de vínculos laborales precarios durante el período. Este aumento es más evidente en la ATS. También se verifica que las regiones Norte y Nordeste, en 2023, presentaron los mayores porcentajes de vínculos laborales precarios de enfermeros en la APS y la ATS.

**Figura 2-:** Distribución espacial del porcentaje (%) de vínculos laborales precarios de enfermeros, según el tipo de vínculo, por nivel de atención a la salud en los municipios de Brasil, 2010, 2016 y 2023

Notas: (i) los municipios en color blanco indican datos faltantes sobre los vínculos profesionales en el CNES y (ii) número de observaciones (n) incluidas en el análisis de distribución espacial, siendo: (ii.1): Atención Primaria de Salud: 105.587 (años 2010, 2016 y 2023); (ii.2): Atención Secundaria de Salud: 103.358 (años 2010, 2016 y 2023); (ii.3): Atención Terciaria de Salud: 148.241 (años 2010, 2016 y 2023)

Los resultados mostraron un aumento de la precarización del trabajo de los enfermeros en Brasil según el tipo de vínculo y la necesidad de implementar estrategias de desprecarización en el SUS.

## Discusión

Los resultados de este estudio indicaron una tendencia creciente de la PVL de enfermeros en todos los niveles de atención a la salud en Brasil, con los mayores incrementos en la ASS y la ATS. En la APS, la tendencia al aumento de la PVL se observó en todas las regiones, excepto en la región Sur. En la ASS y la ATS, hubo un crecimiento de la PVL en todas las regiones del país. Independientemente del nivel de atención, la mayoría de las unidades federativas presentaron una tendencia creciente. En 2023, la APS y la ASS mostraron los mayores porcentajes de vínculos de TP en comparación con la ATS. El análisis espacial evidenció un aumento en el número de municipios con un elevado porcentaje de vínculos de TP de enfermeros, especialmente en la ATS.

La mayoría de las investigaciones que analizó la PVT se centró en estudiar el tipo de vínculo como *proxy* del indicador de TP^(6,30-32)^, un enfoque similar al adoptado en este estudio. En general, los estudios asociaron ciertos tipos de vínculo al TP, dado que presentan bajos niveles de remuneración, reducción de derechos y de protección jurídica del empleo. Entre ellos se destacan: profesionales autónomos con vínculo directo o intermediado por organizaciones de la sociedad civil, entidades filantrópicas o sin fines de lucro, cooperativas, empresas privadas, organizaciones no gubernamentales y organizaciones sociales, además de becarios, profesionales en cargos de confianza, consultores y aquellos con contratos por tiempo determinado^(30-32)^. Otros enfoques, sin embargo, consideraron aspectos multidimensionales para medir la precariedad de los vínculos laborales, incluyendo el uso de escalas amplias^(32–35)^.

Además, no se encontraron investigaciones con análisis de tendencia y el mismo enfoque metodológico para comparación directa con esta investigación. No obstante, los resultados fueron similares a los de un estudio realizado con datos de 2007 a 2021, que mostró un aumento en el porcentaje de vínculos de TP entre profesionales de educación física que actúan en el SUS. Ese estudio identificó que la APS concentró el mayor número de registros de profesionales con vínculos laborales precarios, lo contrario de lo observado en la ASS y la ATS^(6)^. Otra investigación que analizó datos del Programa de Mejora del Acceso y la Calidad de la Atención Básica mostró una alta proporción de enfermeros con vínculos de TP, como contratos temporales o intermediados por Organizaciones Sociales, además de una tendencia de reducción de vínculos estables en la APS^(11)^. Los resultados de este estudio y la evidencia previa sugieren que la PVL en la APS es un problema emergente y creciente en Brasil.

El escenario creciente de la PVL de enfermeros en la APS en el país es motivo de preocupación. La APS actúa como eje orientador del cuidado y modelo prioritario de organización del SUS. Se caracteriza por acciones individuales y colectivas que incluyen la promoción y protección de la salud, la prevención de enfermedades, el diagnóstico, el tratamiento, la rehabilitación y el mantenimiento de la salud^(36)^. El impacto directo de la PVL en la APS es la alta rotación de enfermeros, lo que genera sobrecarga para los demás profesionales, dificulta el establecimiento de vínculos con la población atendida y reduce la calidad asistencial^(11,30)^.

En resumen, el presente estudio mostró un mayor porcentaje de incremento de los vínculos de TP en la ASS y la ATS en comparación con la APS. No se encontraron estudios de tendencia que compararan las variaciones porcentuales según el nivel de atención. Sin embargo, el estudio que analizó la PVL en profesionales de educación física mostró un aumento del número de registros de profesionales con vínculos precarios en la ASS y la ATS en comparación con la APS^(6)^. Algunas hipótesis para este incremento más acentuado en la ASS y la ATS pueden incluir la participación público-privada más reciente en estos niveles de atención, incluyendo el aumento de contratos con Organizaciones Sociales en la atención especializada^(6)^. Además, a partir de 2020, la pandemia por la enfermedad causada por el nuevo coronavirus (COVID-19) exigió nuevas contrataciones por parte de los gestores, ante la sobrecarga de los servicios de salud, especialmente en la atención especializada^(2)^. Este aspecto puede haber contribuido al mayor incremento de la PVL de enfermeros en la atención especializada en comparación con la APS^(15)^.

Se verificó el mayor porcentaje de vínculos de TP de enfermeros en las regiones Norte y Nordeste. Este resultado también fue observado en profesionales de educación física^(6)^. Los estudios muestran que las regiones Norte y Nordeste concentran los municipios más pequeños del país y presentan condiciones de infraestructura sanitaria inferiores en comparación con otras regiones^(22-23)^. Estos factores, entre otros, generan mayores dificultades para atraer y retener profesionales de salud calificados^(37-38)^ y contribuyen a una mayor adopción de formas de contratación precarias en estas regiones^(6)^. Algunos indicadores pueden señalar posibles impactos de la PVL en la retención de profesionales y la atención en salud en estas localidades; por ejemplo, las regiones Norte y Nordeste presentan la menor razón de profesionales de salud por habitante y los menores porcentajes de acceso a los servicios de salud en comparación con las demás regiones^(39-40)^.

El alto porcentaje de vínculos de TP en todos los niveles de atención a la salud y su tendencia creciente siguen representando un problema para el sistema de salud, a pesar de las políticas e iniciativas implementadas por el Ministerio de Salud para su enfrentamiento, como el programa *DesprecarizaSUS* ^(19)^. La tendencia al aumento del porcentaje de vínculos laborales precarios puede estar relacionada con diversos factores, entre ellos los problemas de subfinanciamiento del SUS^(12)^.

Desde una perspectiva operativa, las prácticas de precarización buscan permitir mayor flexibilidad y reducir costos en los servicios de salud^(41)^. Sin embargo, algunos estudios han demostrado que un alto porcentaje de PVL afecta la organización y gestión de los servicios de salud, limita la expansión de la cobertura de acciones y servicios y compromete la calidad e integralidad de la atención, pudiendo generar resultados negativos para los usuarios y reducir el acceso a los servicios^(2,42)^. Además, la mercantilización y la desechabilidad de la fuerza de trabajo en salud, así como los cambios en los modelos de gestión y organización del trabajo ocasionados por la PVL, también generan vulnerabilidad y aumentan los riesgos relacionados con las condiciones de seguridad y salud en el trabajo, exponiendo a los profesionales a diversos daños físicos y mentales. Asimismo, el aislamiento resultante de esta práctica, sumado a la desvalorización profesional, puede afectar negativamente la solidaridad de clase, debilitando la fuerza sindical^(43)^.

Este estudio presentó algunas limitaciones, como la calidad de los datos disponibles en el CNES, lo que puede llevar a una subestimación o sobreestimación del indicador de porcentaje de vínculos laborales precarios. Por otro lado, la base de datos es confiable^(44)^ y es ampliamente utilizada en estudios del área. El estudio se centró en la investigación de la PVL, sin abarcar otras dimensiones de la precarización del trabajo en salud, como la multiplicidad de vínculos, la baja remuneración, la falta de infraestructura para el trabajo y el descanso, la violencia, el acoso, el esfuerzo repetitivo y las consecuencias de la PVL para la salud de los trabajadores y la calidad de la atención a los usuarios^(2,41)^. Además, la base de datos no permitió evaluar el número de vínculos de un mismo enfermero, debido a la ausencia de identificación de los participantes, lo que impidió analizar la tendencia temporal de vínculos dobles o triples de estos profesionales. Por último, esta investigación incluyó datos de antes y durante la pandemia de COVID-19, período en el cual muchos enfermeros fueron contratados para atender las demandas urgentes de salud, en su mayoría mediante contratos temporales y, en ocasiones, sin garantías de derechos sociales. Este hecho puede haber contribuido a reducir la precisión en la estimación de la tendencia temporal.

Sin embargo, este estudio presenta avances importantes en el conocimiento sobre la PVL de enfermeros, al explorar tendencias temporales de esta dimensión desagregadas por regiones, unidades federativas y nivel de atención a la salud, aportando elementos fundamentales para el monitoreo y la formulación de políticas públicas que promuevan la desprecarización del trabajo en el SUS.

Nuevas investigaciones pueden analizar los determinantes de la PVL de enfermeros, como el estudio de variables de naturaleza socioeconómica [por ejemplo, Producto Interno Bruto (PIB) per cápita, salario promedio, gasto en salud per cápita, entre otras], infraestructura (como los tipos de unidades), contribuyendo así a una mejor comprensión del fenómeno de la PVL, especialmente en determinadas regiones y estados de Brasil. Más allá de estos determinantes, es importante entender cómo la precarización afecta ciertos resultados, ya sea en términos de eficiencia de los sistemas y servicios de salud, o en relación con la calidad asistencial. Comprender la precarización de la FTS exige un enfoque multidimensional. Además de los tipos de vínculos analizados en este estudio, futuras investigaciones pueden incorporar otras fuentes secundarias para examinar componentes adicionales, como ingresos medios, multiplicidad de vínculos y enfermedades de los trabajadores, lo que permitiría la construcción de un índice multidimensional de precarización. Existe un amplio cuerpo de evidencia que demuestra los efectos adversos de la precarización en la salud del trabajador sanitario, a nivel individual, como enfermedades y vulnerabilidad socioeconómica. Sin embargo, también es fundamental investigar, a nivel ecológico, cómo la precarización puede influir en factores como la retención, la densidad de profesionales y la calidad de la atención en salud.

## Conclusión

Este estudio mostró una tendencia creciente de la PVL en enfermeros en Brasil, independientemente del nivel de atención y de la región geográfica, con base en el tipo de vínculo. Las regiones Norte y Nordeste presentaron los mayores porcentajes de vínculos de TP, lo que sugiere desigualdades en la PVL en el país. Los resultados mostraron el aumento de la precarización de los vínculos laborales de enfermeros y la necesidad de estrategias de desprecarización en el SUS.

## Data Availability

El conjunto de datos de este artículo está disponible en https://drive.google.com/drive/folders/1rEtK9rJJKjRWVFd6OByr_MPx8m9cxkHe
